# The hidden effects of metabolism-disrupting agents on alcohol-related liver disease risk: an integrated epidemiological, machine learning, and network toxicology Analysis

**DOI:** 10.1186/s12967-026-08673-2

**Published:** 2026-07-25

**Authors:** Longpeng Ma, Jinning Zhang, Juntong Wei, Wenbo Bian, Tao Bo, Shanshan Shao, Xinhua Li, Jiajun Zhao, Zhenyu Yao

**Affiliations:** 1 Key Laboratory of Endocrine Glucose & Lipids Metabolism and Brain Aging, Ministry of Education, Department of Endocrinology, Shandong Provincial Hospital Affiliated to Shandong First Medical University, Jinan, Shandong 250021 China; 2 Shandong Clinical Research Center of Diabetes and Metabolic Diseases, Jinan, Shandong 250021 China; 3 Shandong Institute of Endocrine and Metabolic Diseases, Jinan, Shandong 250021 China; 4 “Chuangxin China” Innovation Base of stem cell and Gene Therapy for endocrine Metabolic diseases, Jinan, Shandong 250021 China; 5 Endocrine and Metabolic Diseases Hospital of Shandong First Medical University, Jinan, Shandong 250021 China

**Keywords:** Alcohol-related liver disease, Metabolism-disrupting agents, Machine learning, Network toxicology, Environmental exposure assessment

## Abstract

**Background:**

Individual susceptibility to alcohol-related liver disease (ALD) varies substantially despite similar alcohol consumption patterns. Emerging evidence suggests metabolism-disrupting agents (MDAs) may synergistically amplify alcohol-induced hepatotoxicity, yet population-level evidence remains limited. This study investigated associations between 40 MDAs and ALD risk using integrated analytical approaches.

**Methods:**

We analyzed 13,472 National Health and Nutrition Examination Survey participants (2005–2016). Forty MDAs spanning per- and polyfluoroalkyl substances, phenolic compounds, phthalate metabolites, polycyclic aromatic hydrocarbon metabolites, and volatile organic compound metabolites were measured using standardized protocols. Multivariable logistic regression assessed MDA-ALD associations. Restricted cubic splines characterized dose-response relationships. Subgroup analyses identified vulnerable populations by sex, age, race/ethnicity, body mass index, and hyperlipidemia status. Machine learning algorithms including LightGBM were developed with nested cross-validation (to prevent data leakage during feature selection) to identify predictive biomarkers. Network toxicology integrated computationally predicted MDA targets with ALD gene expression data (GEO GSE28619), followed by pathway enrichment analyses.

**Results:**

Three MDAs demonstrated robust positive associations: benzylmercapturic acid (BMA; OR: 1.56, 95% CI: 1.31–1.86), perfluorohexanesulfonic acid (PFHxS; OR: 1.56, 95% CI: 1.28–1.90), and benzophenone-3 (BP-3; OR: 1.40, 95% CI: 1.15–1.72). BMA and PFHxS exhibited linear dose-response relationships without thresholds, while ATCA showed an inverted U-shaped pattern and BP-3 displayed a plateau pattern. Phenolic compounds and phthalates demonstrated stronger associations in females. LightGBM achieved optimal performance (cross-validation AUC: 0.760, test AUC: 0.706), identifying N-acetyl-S-(3-hydroxypropyl)-L-cysteine, N-acetyl-S-(N-methylcarbamoyl)-L-cysteine, and mono-carboxyoctyl phthalate as key predictive biomarkers, including compounds not significant in regression. Network analysis identified 192 shared genes with enrichment in neuroactive ligand-receptor interactions and nuclear receptor pathways (PPARα/γ, PXR, FXR).

**Conclusions:**

Specific MDAs demonstrated significant ALD associations through complementary approaches. Evidence suggests MDAs may function as signal disruptors within neuro-endocrine-immune networks, with pathways paralleling alcohol-induced hepatotoxicity. Findings underscore incorporating environmental assessments into ALD risk stratification and highlight needs for prospective validation and mechanistic studies.

**Supplementary information:**

The online version contains supplementary material available at https://doi.org/10.1186/s12967-026-08673-2.

## Background

Alcohol-related liver disease (ALD) encompasses a spectrum of progressive hepatic pathologies from chronic alcohol consumption, including steatosis, hepatitis, fibrosis, cirrhosis, and hepatocellular carcinoma [1]. ALD represents a major global health challenge, with ALD-related mortality contributing to 5.9% of worldwide deaths [2]. As the primary cause of cirrhosis globally, ALD is the leading cause of liver-related deaths in Europe [3, 4]. Despite this burden, therapeutic options remain limited: corticosteroids are the only proven therapy for severe alcoholic hepatitis, effective in 50–60% of cases, with no FDA-approved medications for ALD [5, 6]. The most effective strategy remains sustained abstinence and attenuation of inflammatory responses [7]. However, individual susceptibility varies markedly: only approximately 35% of individuals with alcohol use disorder develop overt ALD [4, 8]. This heterogeneity highlights the need to identify modifiable environmental risk factors that may synergistically modulate ALD pathogenesis.

The paradigm attributing ALD solely to alcohol has evolved with the “multiple-hit” hypothesis, recognizing that liver disease arises from synergistic interactions among concurrent factors including insulin resistance, nutritional status, gut microbiota, and environmental exposures acting on genetically susceptible individuals [9–11]. This shift implies that individual vulnerability extends beyond alcohol quantity to include modifiable environmental factors. Within this framework, environmental chemicals may represent underappreciated cofactors that could synergistically amplify alcohol-induced hepatotoxicity. Environmental exposures have been implicated across the full spectrum of alcohol-related hepatic pathologies—from steatosis and alcoholic hepatitis to fibrosis, cirrhosis, and hepatocellular carcinoma—suggesting that chemical co-exposures may be a critical driver of the substantial heterogeneity in individual ALD susceptibility. Emerging evidence supports this hypothesis: a cohort study of more than 400,000 participants found that PM2.5-associated liver disease risk was more pronounced among alcohol drinkers versus non-drinkers [12]. Similarly, PFAS exposure was associated with alcoholic fatty liver disease among heavy drinkers, but not among light drinkers or abstainers [13]. Experimental validation from a chronic-binge ethanol plus PFOS mouse model demonstrated that PFOS exacerbated alcohol-induced steatosis, elevated aminotransferases, and activated pathways of lipid metabolism, oxidative stress, and fibrosis [14]. These findings suggest that environmental pollutants and alcohol converge on shared mechanisms including lipid dysregulation, oxidative stress, and impaired detoxification [15, 16]. However, the role of environmental chemicals in ALD remains poorly characterized, with limited population-level data on exposure-disease associations [14]. This gap is important given the ubiquity of chemical exposures and potential for synergistic effects in at-risk populations, suggesting that environmental factors may substantially contribute to the ALD burden.

Among environmental exposures that may influence ALD, metabolism-disrupting agents (MDAs) represent an important yet understudied risk factor class. MDAs are ubiquitous contaminants that disrupt metabolic homeostasis through mechanisms including lipid/glucose dysregulation, hormone disruption, and mitochondrial dysfunction [17]. Major MDA classes include PFAS, phenolic compounds, phthalates, PAH metabolites, and VOC metabolites, with widespread human exposure via water, food packaging, personal care products, and air [18–21]. MDA exposure is pervasive: PFAS are detected in >98% of the U.S. population [22], phthalate metabolites in >75% [23], and phenolic compounds including bisphenol A in >90% [24]. These compounds accumulate in the liver and are implicated in metabolic dysfunction-associated steatotic liver disease (MASLD), with evidence linking exposures to steatosis, inflammation, and fibrosis. A 2024 expert consensus established key characteristics of MDAs, including disruption of lipid and glucose homeostasis—properties directly relevant to liver dysfunction [17]. Despite biological plausibility, important research gaps remain regarding MDA-ALD associations. Studies examining single MDAs fail to capture real-world mixture exposures and potential interactions. While MDA-MASLD associations are well-documented [25, 26], large-scale MDA-ALD studies are lacking. The intersection of MDA exposure and alcohol consumption—potentially converging on shared mechanisms of lipid disruption, oxidative stress, and impaired detoxification—remains poorly characterized. Mechanisms underlying MDA-ALD interactions remain unclear, limiting identification of vulnerable populations and prevention strategies.

To address these gaps, we analyzed data from the National Health and Nutrition Examination Survey (NHANES), a nationally representative study with biomonitored exposures and health outcomes. We systematically evaluated associations between 40 MDAs across five chemical classes (PFAS, phenols, phthalates, PAH metabolites, VOC metabolites) and ALD risk. Stratified analyses identified susceptible subpopulations by sex, age, race/ethnicity, BMI, and hyperlipidemia status. Machine learning approaches identified and prioritized MDAs most predictive of ALD risk in a data-driven manner. Network toxicology and pathway enrichment analyses elucidated potential molecular mechanisms linking MDA exposures to ALD pathogenesis. This study combines epidemiological, machine learning, and network toxicology approaches to assess MDA-ALD associations in a population-based cohort. Our findings could inform prevention strategies targeting modifiable environmental contributors to ALD.

## Methods

### Study population

This study included 13,472 participants from four NHANES cycles (2005–2006, 2011–2012, 2013–2014, 2015–2016). NHANES is a nationally representative survey of the noninstitutionalized U.S. population conducted by the Centers for Disease Control and Prevention (CDC). The study protocol was approved by the National Center for Health Statistics (NCHS) Institutional Review Board, and all participants provided written informed consent. Additional information is available at http://www.cdc.gov/nchs/nhanes.

Exclusion criteria included: (1) age < 20 or >85 years, pregnancy, or lactation; (2) missing essential data on anthropometrics (height, weight, waist circumference), alcohol consumption, blood pressure, or laboratory tests (ALT, AST, HDL, glucose, triglycerides); (3) other liver diseases including metabolic dysfunction-associated steatotic liver disease (MASLD), viral hepatitis, or unspecified liver conditions. Participant selection is shown in Figure S1.

### Metabolism-disrupting agent measurement

Forty MDAs spanning five classes were measured: per- and polyfluoroalkyl substances (PFAS, *n* = 3), phenols and preservatives (*n* = 6), phthalate metabolites (*n* = 10), polycyclic aromatic hydrocarbon (PAH) metabolites (*n* = 6), and volatile organic compound (VOC) metabolites (*n* = 15). Detailed chemical names and abbreviations are provided in Table S1.

All MDAs were measured in serum or urine samples using standardized protocols at the CDC Division of Laboratory Sciences. Analytical methods included liquid chromatography-tandem mass spectrometry (LC-MS/MS) with class-specific sample preparation: solid-phase extraction (SPE) coupled with high-performance LC (HPLC) for PFAS (turbo ion spray interface); SPE-HPLC-MS/MS for phenols and PAH metabolites; online SPE with reversed-phase HPLC and electrospray ionization (ESI) for phthalates; and ultra-performance LC (UPLC)-ESI-MS/MS for VOC metabolites. Detailed protocols are available in NHANES laboratory documentation. Concentrations below the limit of detection (LOD) were imputed as LOD/√2. To ensure data reliability and minimize imputation bias at the lower extreme, MDAs with >30% of measurements below the LOD were excluded prior to analysis.

### Alcohol-related liver disease ascertainment

ALD was defined using a validated composite algorithm based on prior NHANES studies [27, 28]. Participants were classified as having ALD based on: (1) self-reported physician diagnosis of ALD, or (2) elevated aminotransferases (AST or ALT > 25 U/L in women, >35 U/L in men) combined with heavy alcohol consumption (≥3 drinks/day for women, ≥4 for men, or ≥ 5 binge drinking episodes/month), AST/ALT ratio > 1, and exclusion of viral hepatitis B/C and MASLD. MASLD was excluded based on the presence of metabolic dysfunction (BMI ≥ 25 kg/m^2^ or ≥ 2 cardiometabolic risk factors) in individuals without significant alcohol use, following established diagnostic criteria.

### Covariates

Based on prior research, we selected potential confounding factors that could influence the association between MDAs and ALD as covariates, including age, gender, race, educational level, body mass index (BMI), family income-to-poverty ratio (PIR), smoking status, hypertension, and hyperlipidemia. Age was analyzed continuously and categorically (20–39, 40–59, ≥60 years). Race/ethnicity included Non-Hispanic White, Non-Hispanic Black, Mexican American, and Other. Education was categorized as less than high school, high school, or college/above. BMI was dichotomized as < 25 or ≥25 kg/m^2^.

### Statistical analysis

#### Baseline analysis

Descriptive analyses compared baseline characteristics between participants with and without ALD. we analyzed continuous and categorical variables with the t-test and the chi-square test, respectively, reporting mean and standard deviation for continuous variables and frequency (n) and percentage (%) for categorical variables.

#### Association Analysis

To account for urinary dilution, we adjusted urinary MDA concentrations by creatinine, calculated as MDA concentration (μg/L) divided by urinary creatinine concentration (mg/dL), yielding creatinine-corrected concentrations in μg/g creatinine. Serum PFAS concentrations required no creatinine adjustment. All MDA concentrations were log₁₀-transformed to approximate normal distributions and reduce the influence of extreme values. To further minimize the impact of outliers, we applied winsorization at the 1st and 99th percentiles, replacing values below the 1st percentile with the 1st percentile value and values above the 99th percentile with the 99th percentile value. While this robust approach prevents isolated extreme concentrations from driving spurious associations, it inherently yields statistically conservative risk estimates at the upper exposure extremes. For regression and machine learning analyses, log-transformed and winsorized concentrations were standardized to Z-scores (mean = 0, SD = 1) using the formula: Z = (X - mean)/SD. Standardization facilitates comparison of effect sizes across MDAs measured in different units and concentration ranges.

Logistic regression models were used to examine the association between log-transformed MDAs and ALD, and the strength of the association was determined with the odds ratio (OR) and confidence interval (CI). Three regression models were utilized as follows: Model 1 was unadjusted for covariates; Model 2 enhanced Model 1 by including age, sex, race/ethnicity; and Model 3 further augmented Model 2 by integrating all covariates. To further investigate the dose-response relationship between MDAs and ALD, we employed a restricted cubic spline (RCS) model using the R package “rcssci.” Four knots were set at the 5th, 35th, 65th, and 95th percentiles in the models, respectively. In addition, we conducted subgroup analyses of log-transformed MDAs and examined their interactions with age, sex, race/ethnicity, BMI, and hyperlipidemia. Given the exploratory nature of this study utilizing multiple complementary analytical approaches, formal multiple testing correction was not applied as the primary analysis strategy. However, to account for the risk of false-positive findings derived from evaluating 40 MDAs simultaneously, a post-hoc sensitivity analysis was conducted using a highly conservative Bonferroni correction, setting an adjusted significance threshold at α = 0.05/40 = 0.00125.

#### Machine learning model development

##### Feature selection and model construction

The dataset was randomly split 7:3 into training and test sets with stratified sampling. To prevent data leakage, we implemented nested cross-validation with feature selection performed independently within each fold.

In each of the 5 training folds, least absolute shrinkage and selection operator (LASSO) regression (L1 regularization, λ via internal CV) and Random Forest (RF; 1000 trees, Gini importance) independently selected features from the 40 MDAs and covariates (Figure S2). Variables selected by both methods were retained (intersection approach). Nine algorithms were trained with grid-search hyperparameter optimization within each fold: Logistic Regression (LR), Elastic Net (ENET), Decision Tree (DT), Random Forest (RF), Extreme Gradient Boosting (XGBoost), Support Vector Machine with radial basis function kernel (SVM-RBF), Multi-Layer Perceptron (MLP), Light Gradient Boosting Machine (LightGBM), and K-Nearest Neighbors (KNN).

The final model used LightGBM trained on the full training set with 14 consensus features (NPMA, BMA, AMCC, MCOP, HHPM, ATCA, 1-OHPhe, MEOHP, HPMI, 2-OHN, age, BMI, sex, hyperlipidemia) and optimal hyperparameters (learning_rate = 0.05, num_leaves = 31, max_depth = 10). The test set remained completely isolated from feature selection and training procedures.

##### Model evaluation and selection

Model performance was assessed using: (1) discrimination via area under the receiver operating characteristic curve (AUC); (2) calibration via calibration plots comparing predicted versus observed probabilities; and (3) clinical utility via decision curve analysis (DCA) quantifying net benefit across decision thresholds. The algorithm with optimal performance across these metrics was validated on the held-out test set.

##### Model interpretation

SHapley Additive exPlanations (SHAP) analysis interpreted the final model. SHAP values quantify each feature’s contribution to individual predictions based on cooperative game theory. Summary bar plots ranked features by mean absolute SHAP value (overall importance). Beeswarm plots displayed the distribution of SHAP values, with point position indicating contribution magnitude and color representing feature value (red = high, blue = low).

### Network toxicology Analysis

#### Target identification and gene expression analysis

MDA protein targets were identified using Swiss Target Prediction (http://swisstargetprediction.ch/) and Similarity Ensemble Approach (https://sea.bkslab.org/) databases. Duplicate targets were removed after merging.

ALD-related gene expression data were obtained from Gene Expression Omnibus (GEO) dataset GSE28619, which included liver tissue samples from 7 healthy controls and 15 ALD patients profiled on Affymetrix Human Genome U133 Plus 2.0 Arrays. Differential expression analysis using the limma package identified differentially expressed genes (DEGs) at thresholds of *p* < 0.05 and |log₂ fold-change| >1.2.

Weighted gene co-expression network analysis (WGCNA) identified gene modules correlated with ALD. Key steps included: sample clustering to detect outliers; soft-thresholding power selection (β) to achieve scale-free topology (R^2^ > 0.85); topological overlap matrix (TOM) construction; hierarchical clustering with dynamic tree cutting to define modules; and module-trait correlation analysis to identify ALD-associated modules.

Shared genes between MDA targets and ALD-related genes (DEGs plus WGCNA module genes) were identified as candidate mechanistic mediators.

#### Functional enrichment analysis

Gene Ontology (GO) enrichment (Biological Process, Cellular Component, Molecular Function) and Kyoto Encyclopedia of Genes and Genomes (KEGG) pathway enrichment analyses were performed on shared genes using the clusterProfiler R package. Significance was defined as adjusted *p* < 0.05 (Benjamini-Hochberg correction).

### Software and statistical significance

All analyses used R version 4.4.0. Key packages included tidymodels, randomForest, glmnet, xgboost, caret, iml (SHAP), WGCNA, limma, clusterProfiler, and ggplot2. Two-sided *p* < 0.05 indicated statistical significance unless otherwise specified.

## Results

### Participant characteristics

Among 13,472 participants, 6,164 (45.7%) had ALD. The ALD group exhibited distinct demographic and metabolic characteristics (Table 1). Males comprised two-thirds of ALD cases versus 38% of controls (*p* < 0.001). Paradoxically, ALD participants were younger (mean age 46.8 vs. 49.2 years) but had higher BMI (29.8 vs. 27.1 kg/m^2^). The ALD group demonstrated a significantly greater burden of cardiometabolic risk factors, with elevated prevalence of hyperlipidemia (73.7% vs. 62.8%) and hypertension (41.1% vs. 37.5%). Current smoking was also more prevalent in ALD cases (23.0% vs. 19.4%; all *p* < 0.001).

**Table 1:** Baseline characteristics of study participants stratified by alcohol-related liver disease status

	Overall	ALD		p-value
		No	Yes	
**N**	13,472	7308	6164	
**Gender,n(%)**				
Male	6977 (51.8)	2806 (38.4)	4171 (67.7)	<0.001
Female	6673 (48.2)	4502 (61.6)	1993 (32.3)	
**Age**				
mean(SD)	48.13 (17.75)	49.22 (18.96)	46.83 (16.11)	<0.001
**Race/ethnicity,n(%)**				
Non-Hispanic White	5646 (41.9)	2853 (39.0)	2793 (45.3)	<0.001
Non-Hispanic Black	2881 (21.4)	1799 (24.6)	1082 (17.6)	
Mexican American	1970 (14.6)	936 (12.8)	1034 (16.8)	
Other	2975 (22.1)	1720 (23.5)	1255 (20.4)	
**BMI**				
mean(SD)	28.31 (6.80)	27.05 (6.75)	29.80 (6.55)	<0.001
**Education level,n(%)**				
Less than high school	2940 (21.8)	1767 (24.2)	1173 (19.0)	<0.001
High school or equal	2941 (21.8)	1589 (21.7)	1352 (21.9)	
College or above	7591 (56.3)	3952 (54.1)	3639 (59.0)	
**PIR**				
mean(SD)	2.61 (1.65)	2.45 (1.62)	2.81 (1.66)	<0.001
**Hyperlipidemia,n(%)**				
No	4340 (32.2)	2720 (37.2)	1600 (26.3)	<0.001
Yes	9132 (67.8)	4588 (62.8)	4544 (73.7)	
**Hypertension,n(%)**				
No	8200 (60.9)	4570 (62.5)	3630 (58.6)	<0.001
Yes	5272 (39.1)	2738 (37.5)	2534 (41.1)	
**Smoke,n(%)**				
No	10641 (79.0)	5892 (80.6)	4749 (77.0)	<0.001
Yes	2831 (21.0)	1416 (19.4)	1415 (23.0)	

Demographic, anthropometric, and clinical characteristics of 13472 participants from the National Health and Nutrition Examination Survey (NHANES) cycles 2005–2006, 2011–2012, 2013–2014, and 2015–2016, stratified by alcohol-related liver disease (ALD) status. Data are presented as mean (standard deviation [SD]) for continuous variables and n (%) for categorical variables. Continuous variables were compared using independent samples t-tests; categorical variables were compared using weighted chi-square tests. All analyses incorporated NHANES sampling weights to generate nationally representative estimates

Variable definitions: Hyperlipidemia was defined as elevated lipid profiles (total cholesterol, triglycerides, or LDL cholesterol exceeding clinical thresholds) or self-reported current use of lipid-lowering medications. Hypertension was defined as systolic blood pressure ≥ 130 mmHg or diastolic blood pressure ≥ 80 mmHg, or current use of antihypertensive medication, based on the 2017 ACC/AHA guideline. Current smoking was defined as having smoked at least 100 cigarettes in lifetime and currently smoking cigarettes (either every day or some days). BMI, body mass index; PIR, family income-to-poverty ratio. Statistical significance was set at two-sided *p* < 0.05

### Associations between MDAs and ALD

To characterize the relationships between individual MDA exposures and ALD risk, we employed logistic regression models followed by dose-response analysis.

#### Logistic regression Analysis

We first applied fully adjusted logistic regression models to identify MDAs significantly associated with ALD risk across the five chemical classes (Table 2, Table S2). This analysis revealed chemical-specific associations with varying effect magnitudes.

**Table 2:** Associations between metabolism-disrupting agents and alcohol-related liver disease risk across exposure quartiles

		Model 1 (OR 95%CI)	P	Model 2 (OR 95%CI)	P	Model 3 (OR 95%CI)	P
**PFAS (Log ng/mL)**							
PFHxS							
	Q1	Ref.					
	Q2	1.61(1.36,1.91)	<0.0001	1.29(1.08,1.55)	0.01	1.32(1.09,1.60)	0.005
	Q3	1.95(1.65,2.31)	<0.0001	1.39(1.16,1.68)	<0.001	1.44(1.18,1.76)	<0.001
	Q4	2.24(1.89,2.66)	<0.0001	1.55(1.28,1.87)	<0.0001	1.56(1.28,1.90)	<0.0001
PFNA							
	Q1	Ref.					
	Q2	1.27(1.08,1.50)	0.005	1.16(0.97,1.38)	0.11	1.17(0.97,1.41)	0.1
	Q3	1.33(1.13,1.57)	<0.001	1.20(1.00,1.43)	0.04	1.19(0.98,1.44)	0.07
	Q4	1.35(1.14,1.60)	<0.001	1.27(1.05,1.53)	0.01	1.29(1.06,1.57)	0.01
**Phenols and Parabens (Log ng/mg CR)**							
BP-3							
	Q1	Ref.					
	Q2	1.19(1.00,1.41)	0.05	1.33(1.11,1.60)	0.002	1.27(1.04,1.53)	0.02
	Q3	1.26(1.07,1.50)	0.01	1.47(1.22,1.76)	<0.0001	1.35(1.11,1.64)	0.003
	Q4	1.08(0.91,1.28)	0.4	1.49(1.23,1.80)	<0.0001	1.40(1.15,1.72)	0.001
MBP							
	Q1	Ref.					
	Q2	0.87(0.73,1.03)	0.10	1.15(0.96,1.38)	0.13	1.13(0.93,1.36)	0.22
	Q3	0.72(0.60,0.85)	<0.001	1.15(0.95,1.38)	0.15	1.13(0.93,1.38)	0.21
	Q4	0.56(0.47,0.67)	<0.0001	1.14(0.94,1.39)	0.18	1.24(1.01,1.52)	0.04
PPB							
	Q1	Ref.					
	Q2	0.83(0.70,0.99)	0.03	1.09(0.91,1.30)	0.36	1.09(0.90,1.32)	0.37
	Q3	0.73(0.62,0.87)	<0.001	1.29(1.07,1.56)	0.01	1.28(1.05,1.57)	0.01
	Q4	0.51(0.43,0.61)	<0.0001	1.11(0.91,1.35)	0.3	1.16(0.94,1.43)	0.16
2,4-DCP							
	Q1						
	Q2	1.22(1.03,1.44)	0.02	1.27(1.06,1.52)	0.01	1.22(1.01,1.47)	0.04
	Q3	0.95(0.80,1.12)	0.52	0.99(0.83,1.19)	0.91	0.97(0.80,1.17)	0.76
	Q4	0.86(0.73,1.02)	0.09	0.98(0.82,1.18)	0.83	1.01(0.83,1.23)	0.92
**Phthalate Metabolites (Log ng/mg CR)**							
MEP							
	Q1	Ref.					
	Q2	1.02(0.86,1.21)	0.78	1.16(0.97,1.39)	0.11	1.16(0.96,1.40)	0.12
	Q3	0.90(0.76,1.07)	0.23	1.09(0.91,1.30)	0.37	1.03(0.85,1.25)	0.74
	Q4	1.03(0.87,1.22)	0.71	1.30(1.09,1.57)	0.005	1.29(1.07,1.57)	0.01
MiBP							
	Q1	Ref.					
	Q2	1.18(1.00,1.40)	0.06	1.19(0.99,1.42)	0.06	1.16(0.96,1.41)	0.12
	Q3	1.22(1.03,1.44)	0.02	1.28(1.07,1.53)	0.01	1.23(1.01,1.48)	0.04
	Q4	0.85(0.71,1.00)	0.05	0.98(0.82,1.19)	0.87	0.97(0.79,1.18)	0.73
MEOHP							
	Q1	Ref.					
	Q2	1.01(0.85,1.19)	0.95	0.97(0.81,1.15)	0.7	0.90(0.75,1.09)	0.27
	Q3	0.91(0.77,1.08)	0.30	0.93(0.78,1.11)	0.42	0.82(0.68,0.99)	0.04
	Q4	0.85(0.72,1.00)	0.06	0.92(0.77,1.10)	0.38	0.88(0.72,1.06)	0.18
**PAH Metabolites (Log ng/mg CR)**							
1-OHPhe							
	Q1	Ref.					
	Q2	1.01(0.85,1.19)	0.95	1.21(1.02,1.42)	0.03	1.24(1.04,1.48)	0.01
	Q3	0.91(0.77,1.08)	0.3	1.19(1.01,1.41)	0.04	1.20(1.00,1.43)	0.05
	Q4	0.85(0.72,1.00)	0.06	1.14(0.96,1.35)	0.13	1.14(0.95,1.37)	0.16
**VOC Metabolites (Log ng/mg CR)**							
AMCC							
	Q1	Ref.					
	Q2	1.25(1.07,1.46)	0.004	1.24(1.05,1.45)	0.01	1.17(0.99,1.39)	0.07
	Q3	1.19(1.02,1.39)	0.03	1.15(0.97,1.35)	0.1	1.15(0.96,1.38)	0.13
	Q4	1.25(1.07,1.46)	0.004	1.22(1.04,1.44)	0.02	1.31(1.06,1.62)	0.01
ATCA							
	Q1	Ref.					
	Q2	1.08(0.92,1.26)	0.34	1.22(1.04,1.44)	0.01	1.23(1.03,1.46)	0.02
	Q3	1.04(0.89,1.22)	0.58	1.39(1.17,1.63)	<0.001	1.42(1.19,1.69)	<0.0001
	Q4	0.76(0.65,0.89)	<0.001	1.24(1.04,1.47)	0.02	1.24(1.03,1.49)	0.02
BMA							
	Q1	Ref.					
	Q2	1.22(1.05,1.43)	0.01	1.26(1.07,1.49)	0.01	1.20(1.01,1.42)	0.04
	Q3	1.28(1.10,1.50)	0.002	1.38(1.17,1.63)	<0.001	1.28(1.08,1.52)	0.01
	Q4	1.30(1.12,1.52)	<0.001	1.64(1.38,1.93)	<0.0001	1.56(1.31,1.86)	<0.0001
NPMA							
	Q1	Ref.					
	Q2	1.24(1.06,1.44)	0.01	1.29(1.09,1.52)	0.002	1.20(1.01,1.43)	0.04
	Q3	1.36(1.16,1.59)	<0.001	1.38(1.17,1.63)	<0.001	1.24(1.04,1.48)	0.02
	Q4	1.11(0.95,1.30)	0.18	1.17(0.99,1.38)	0.06	1.04(0.87,1.24)	0.7
3/4-MHA							
	Q1	Ref.					
	Q2	1.17(1.00,1.37)	0.04	1.10(0.94,1.30)	0.23	1.09(0.91,1.29)	0.34
	Q3	1.16(1.00,1.36)	0.06	1.04(0.88,1.23)	0.63	1.12(0.94,1.35)	0.21
	Q4	1.27(1.09,1.49)	0.002	1.14(0.96,1.34)	0.13	1.28(1.05,1.56)	0.01
MHBMA3							
	Q1	Ref.					
	Q2	1.00(0.86,1.17)	0.97	0.92(0.78,1.08)	0.33	0.89(0.75,1.06)	0.19
	Q3	0.94(0.80,1.10)	0.43	0.83(0.71,0.98)	0.03	0.77(0.64,0.92)	0.01
	Q4	0.94(0.80,1.09)	0.41	0.82(0.69,0.96)	0.02	0.75(0.59,0.94)	0.01

Odds ratios (OR) and 95% confidence intervals (CI) for the association between metabolism-disrupting agent (MDA) exposure quartiles and alcohol-related liver disease (ALD) risk in 13472 NHANES participants (2005–2006, 2011–2012, 2013–2014, 2015–2016). MDA concentrations were log₁₀-transformed, winsorized at the 1st and 99th percentiles, and categorized into quartiles (Q1–Q4), with the lowest quartile (Q1) serving as the reference group. Three logistic regression models were constructed: Model 1 (unadjusted); Model 2 (adjusted for age, sex, and race/ethnicity); Model 3 (fully adjusted for age, sex, race/ethnicity, body mass index [BMI], family income-to-poverty ratio [PIR], education level, smoking status, hypertension, and hyperlipidemia). Urinary MDA concentrations were creatinine-corrected (ng/mg creatinine [CR]); serum PFAS concentrations are reported in ng/mL. Results for selected MDAs demonstrating significant associations in Model 3 are presented; complete results for all 40 MDAs are available in Table S2. Statistical significance was set at two-sided *p* < 0.05

VOC metabolites exhibited the strongest associations overall. BMA demonstrated the most pronounced effect (Q4 OR: 1.56, 95% CI: 1.31–1.86; P-trend < 0.0001), with risk increasing monotonically across exposure quartiles. AMCC (Q4 OR: 1.31, 95% CI: 1.06–1.62) and ATCA (Q4 OR: 1.24, 95% CI: 1.03–1.49) showed comparable positive associations. Among PFAS, PFHxS demonstrated a robust positive association, with the highest exposure quartile conferring 56% increased ALD risk (Q4 OR: 1.56, 95% CI: 1.28–1.90; P-trend < 0.0001), while PFNA showed a more modest effect (Q4 OR: 1.29, 95% CI: 1.06–1.57; P-trend = 0.01). Phenolic compounds BP-3 (Q4 OR: 1.40, 95% CI: 1.15–1.72), MBP (OR: 1.24, 95% CI: 1.01–1.52), and PPB (OR: 1.28, 95% CI: 1.05–1.57) demonstrated significant positive associations. Importantly, as a rigorous sensitivity check for multiple comparisons, the robust positive associations observed for BMA, PFHxS, and BP-3 remained highly statistically significant even when evaluated against the stringent Bonferroni-adjusted threshold (*p* < 0.00125).

Notably, MHBMA3 (a 1,3-butadiene metabolite) exhibited a significant inverse association (Q4 OR: 0.75, 95% CI: 0.59–0.94; P-trend = 0.01), suggesting potential protective effects or confounding by individual detoxification capacity. However, this inverse association should be interpreted with caution given the cross-sectional design, as it may reflect reverse causation, residual confounding, or MHBMA3 functioning as a marker of robust Phase II detoxification capacity rather than a causal protective factor; these possibilities are examined in detail in Section “Chemical-specific associations and dose-response Characteristics”. Complete results for all 40 MDAs are provided in Table S2.

#### Dose-response relationships between MDAs and ALD risk

**Fig. 1:**
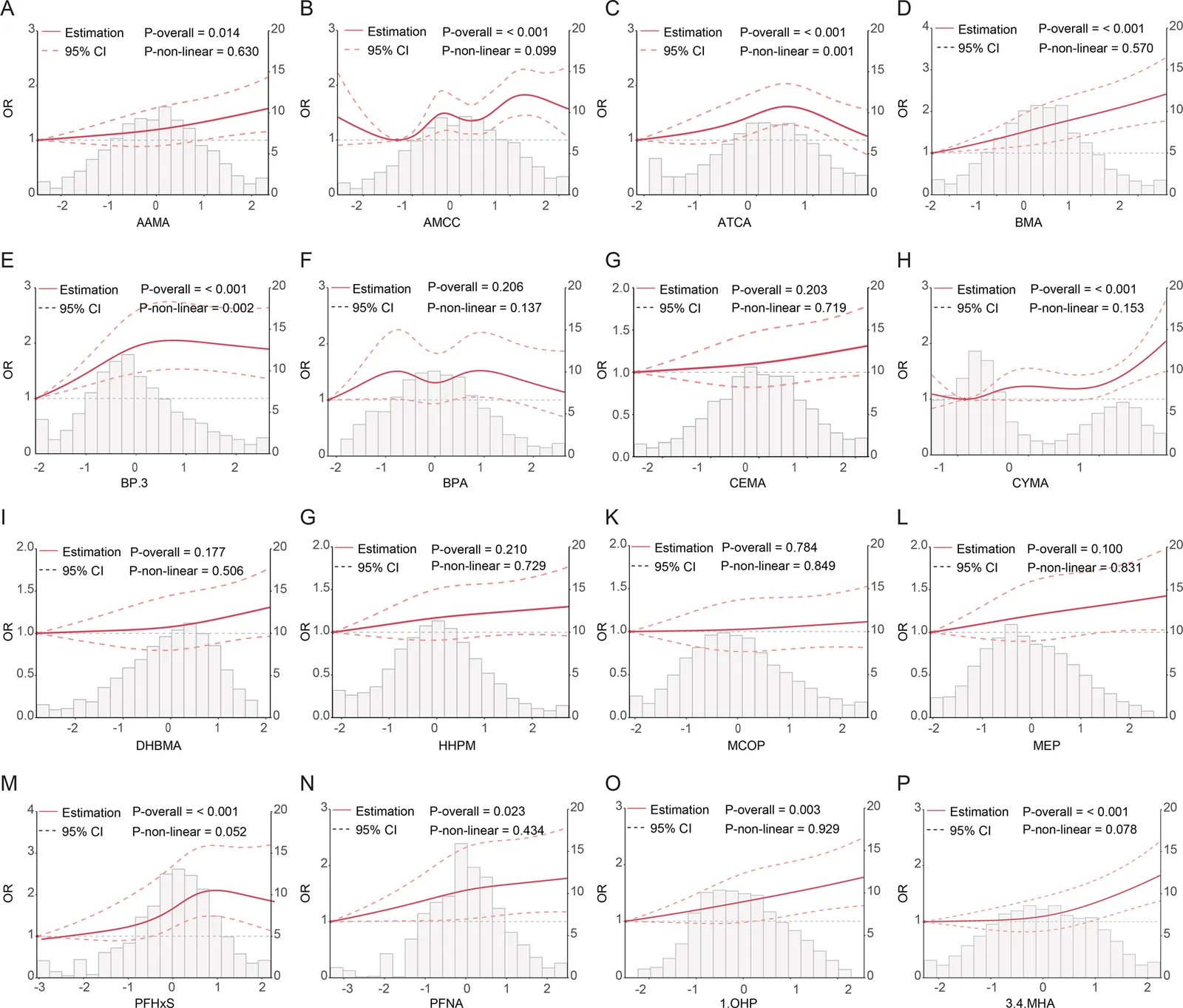
Dose-response relationships between selected metabolism-disrupting agents and alcohol-related liver disease risk. Restricted cubic spline (RCS) curves illustrating dose-response associations between 16 selected metabolism-disrupting agent (MDA) exposures and alcohol-related liver disease (ALD) risk in 13,472 NHANES participants (2005–2006, 2011–2012, 2013–2014, 2015–2016). MDA concentrations were log₁₀-transformed, winsorized at the 1st and 99th percentiles, standardized to Z-scores (mean = 0, SD = 1), and modeled continuously using RCS with four knots positioned at the 5th, 35th, 65th, and 95th percentiles of the standardized exposure distribution. The reference level (OR = 1.0) was set at the 10th percentile of each MDA’s distribution. Logistic regression models were adjusted for age, sex, race/ethnicity, body mass index (BMI), family income-to-poverty ratio (PIR), education level, smoking status, hypertension, and hyperlipidemia, incorporating NHANES sampling weights to generate nationally representative estimates. The x-axis represents standardized MDA concentrations (Z-scores); the y-axis represents odds ratios (OR) for ALD risk. Solid lines represent point estimates of ORs, and shaded areas indicate 95% confidence intervals. Histograms at the bottom of each panel display the distribution of standardized MDA concentrations across the study population. P-overall indicates statistical significance of the overall association; P-nonlinear tests for departure from linearity derived from Wald tests of the cubic spline terms. Original urinary MDA concentrations were creatinine-corrected (ng/mg creatinine); serum PFAS concentrations were in ng/mL prior to log-transformation and standardization

To further characterize the nature of these associations, we employed restricted cubic spline (RCS) analysis to examine dose-response patterns for 16 key MDAs (Fig. 1). This analysis revealed heterogeneity in exposure-response shapes, with implications for risk assessment.

Most MDAs demonstrated linear relationships without evidence of thresholds. PFHxS (P-overall < 0.001, P-non-linear = 0.052), BMA (P-overall < 0.001, P-non-linear = 0.570), CYMA (P-overall < 0.001, P-non-linear = 0.153), and 1-OHP (P-overall < 0.001, P-non-linear = 0.929) showed monotonic risk increases across the full exposure range, suggesting no safe lower limit for these compounds. The high P-non-linear values indicate that linear models adequately captured these dose-response relationships, with no significant improvement from allowing non-linear terms.

In contrast, ATCA and BP-3 exhibited distinct non-linear patterns. ATCA displayed a pronounced inverted U-shaped curve (P-overall < 0.001, P-non-linear = 0.001), with ALD risk increasing progressively at low-to-moderate exposure levels, reaching a peak at approximately the median-to-75th percentile range, and subsequently attenuating at higher concentrations. This non-monotonic pattern suggests that moderate ATCA exposures confer the greatest hepatotoxic risk, while the attenuation at higher levels may reflect activation of compensatory detoxification mechanisms or survivor bias in the study population. BP-3 exhibited a non-linear pattern (P-overall < 0.001, P-non-linear = 0.002) characterized by an initial positive association at low-to-moderate exposures that plateaued at higher concentration levels. This plateau effect suggests that the exposure-response relationship may be constrained at higher doses, potentially due to receptor saturation or activation of compensatory mechanisms.

MCOP, BPA, and MEP showed no significant associations within the studied exposure ranges (all P-overall > 0.10), with RCS curves remaining close to the null value across their distributions and wide confidence intervals encompassing no effect. RCS curves for the other 24 MDAs are provided in Figure S3.

### Effect modification by demographic and metabolic factors

Subgroup analyses stratified by sex, age, race, BMI, and hyperlipidemia status revealed significant heterogeneity in MDA-ALD associations.

Sex-stratified analyses revealed pronounced effect modification (Table 3). Among males, PFHxS (OR: 1.18, 95% CI: 1.08–1.29; P-interaction = 0.02), AMCC, and BMA showed significant positive associations, while 2,4-DCP and 2,5-DCP exhibited protective effects (both OR ≈0.90). In contrast, phenolic compounds (BP-3, MCNP, MCOP) and phthalates showed significantly stronger associations among females (all P-interaction < 0.01). These stronger female associations for lipophilic endocrine disruptors likely reflect estrogen-mediated regulation of hepatic lipid partitioning, greater toxicant sequestration in higher baseline adipose tissue mass, and sex-specific differences in basal alcohol detoxification capacities. The inverse association of MHBMA3 was female-specific (OR: 0.90, 95% CI: 0.83–0.97; P-interaction = 0.04).

**Table 3:** Sex-stratified associations between metabolism-disrupting agents and alcohol-related liver disease risk

Gender	Male		Female		
Variable	OR(CI)	*p* value	OR(CI)	*p* value	P-int
**PFAS**					
PFeDA	0.941 (0.858–1.031)	0.19	0.964 (0.884–1.05)	0.40	0.71
PFHxS	1.179 (1.075–1.293)	**0.00**	1.148 (1.044–1.263)	**0.01**	0.70
PFNA	1.019 (0.933–1.114)	0.67	1.073 (0.981–1.174)	0.13	0.43
**PHEN**					
BP3	1.069 (0.978–1.168)	0.14	1.446 (1.309–1.599)	**0.00**	**0.00**
BPA	0.992 (0.905–1.087)	0.86	1.07 (0.981–1.167)	0.13	0.24
MBP	0.994 (0.9–1.099)	0.91	1.026 (0.937–1.123)	0.58	0.65
PPB	0.997 (0.9–1.104)	0.95	1.07 (0.975–1.174)	0.16	0.32
24DCP	0.897 (0.818–0.983)	**0.02**	0.993 (0.909–1.086)	0.88	0.12
25DCP	0.896 (0.818–0.98)	**0.02**	0.984 (0.9–1.077)	0.73	0.15
**PHTH**					
MCNP	0.96 (0.875–1.053)	0.39	1.196 (1.097–1.305)	**0.00**	**0.00**
MCOP	0.985 (0.898–1.08)	0.75	1.181 (1.083–1.289)	**0.00**	**0.01**
MECPP	0.951 (0.867–1.043)	0.29	1.094 (1.004–1.194)	**0.04**	**0.03**
MnBP	0.971 (0.889–1.061)	0.51	0.953 (0.871–1.043)	0.30	0.78
MCPP	0.934 (0.851–1.025)	0.15	1.11 (1.019–1.211)	**0.02**	**0.01**
MEP	1.021 (0.93–1.12)	0.66	1.042 (0.957–1.136)	0.34	0.75
MEHHP	0.971 (0.885–1.064)	0.53	1.073 (0.984–1.17)	0.11	0.12
MiBP	0.96 (0.878–1.049)	0.37	1.007 (0.921–1.101)	0.88	0.45
MEOHP	0.922 (0.84–1.011)	0.09	1.027 (0.942–1.119)	0.55	0.10
MBzP	0.937 (0.855–1.026)	0.16	0.978 (0.896–1.067)	0.62	0.50
**PAH**					
1.OHN	0.948 (0.87–1.033)	0.23	0.975 (0.899–1.056)	0.53	0.65
2.OHN	0.959 (0.88–1.044)	0.33	1.114 (1.029–1.208)	**0.01**	**0.01**
2.OHF	0.941 (0.862–1.028)	0.18	1.037 (0.958–1.122)	0.37	0.11
3.OHF	0.948 (0.867–1.037)	0.24	1.014 (0.938–1.096)	0.73	0.27
1.OHPHE	0.976 (0.895–1.065)	0.59	1.17 (1.08–1.267)	**0.00**	**0.00**
1.OHP	0.946 (0.865–1.035)	0.23	1.137 (1.052–1.229)	**0.00**	**0.00**
**VOC Metabolites**					
AAMA	0.968 (0.889–1.055)	0.46	1.103 (1.021–1.191)	**0.01**	**0.03**
AMCC	1.099 (1.01–1.195)	**0.03**	1.047 (0.969–1.131)	0.25	0.41
ATCA	0.975 (0.889–1.069)	0.59	1.169 (1.081–1.264)	**0.00**	**0.00**
BMA	1.144 (1.052–1.245)	**0.00**	1.191 (1.101–1.29)	**0.00**	0.49
NPMA	0.959 (0.879–1.045)	0.34	1.167 (1.08–1.261)	**0.00**	**0.00**
CEMA	1.025 (0.944–1.112)	0.56	1.008 (0.931–1.092)	0.84	0.78
CYMA	1.014 (0.928–1.107)	0.76	0.99 (0.918–1.067)	0.79	0.69
DHBMA	1.001 (0.921–1.088)	0.99	1.088 (1.006–1.176)	**0.03**	0.15
HPMA	0.959 (0.884–1.041)	0.32	0.957 (0.883–1.037)	0.28	0.97
HHPM	1.005 (0.926–1.091)	0.91	1.053 (0.972–1.14)	0.21	0.42
2.MHA	1.03 (0.946–1.122)	0.50	1.054 (0.977–1.138)	0.18	0.69
3.4.MHA	1.03 (0.946–1.12)	0.50	1.087 (1.006–1.175)	**0.04**	0.35
MHBMA3	0.963 (0.885–1.048)	0.38	0.899 (0.832–0.972)	**0.01**	0.24
PGA	0.969 (0.889–1.057)	0.47	1.031 (0.956–1.111)	0.43	0.29
HPMI	0.994 (0.913–1.081)	0.89	0.902 (0.835–0.974)	**0.01**	0.10

Odds ratios (OR) and 95% confidence intervals (CI) for associations between metabolism-disrupting agent (MDA) exposures and alcohol-related liver disease (ALD) risk, stratified by sex. MDA concentrations were log₁₀-transformed, winsorized at the 1st and 99th percentiles, and standardized to Z-scores (mean = 0, SD = 1); ORs represent the change in ALD risk per 1-standard deviation increase in MDA concentration. Logistic regression models were adjusted for age, race/ethnicity, body mass index (BMI), family income-to-poverty ratio (PIR), education level, smoking status, hypertension, and hyperlipidemia. All models incorporated NHANES sampling weights to account for the complex survey design. P-int represents the *p*-value for the multiplicative interaction term (MDA × sex) derived from Wald tests of the interaction coefficient in logistic regression models. Urinary MDA concentrations were creatinine-corrected (ng/mg creatinine [CR]); serum PFAS concentrations are reported in ng/mL. Bold *p*-values indicate statistical significance (*p* < 0.05, two-sided)

Age, race, BMI, and hyperlipidemia status further modified MDA-ALD associations (Tables S3–S6). PFHxS associations remained robust across all age groups, whereas BP-3 and MBP effects varied significantly with age (P-interaction < 0.05). Race-stratified analyses identified differential susceptibility patterns, with stronger PFNA and MCNP associations among Non-Hispanic Whites. Individuals with hyperlipidemia showed amplified VOC effects, suggesting that pre-existing dyslipidemia critically compromises basal hepatic lipid handling, thereby lowering the threshold for VOC-induced lipotoxicity. Regarding heavy drinkers specifically, while fully stratified higher-order interaction analyses (e.g., heavy drinking status × MDA exposure × covariates) were precluded by sample size constraints within these highly partitioned sub-strata, the biological plausibility of extreme synergistic toxicity is strongest in this subgroup. Concurrent heavy alcohol and MDA exposure aggressively exhaust shared hepatic detoxification systems (e.g., CYP2E1, glutathione) that are already strained by baseline metabolic dysfunction.

### Machine learning-based risk prediction

While traditional regression identified individual MDA-ALD associations, machine learning approaches can capture complex non-linear relationships and interactions that may enhance risk prediction. To explore this potential, we developed predictive models using nine algorithms with nested cross-validation.

#### Feature selection and model performance

We systematically compared nine machine learning algorithms via 5-fold cross-validation, with independent feature selection performed in each fold to prevent data leakage (Table S7). Among these, LightGBM achieved optimal performance.

**Fig. 2:**
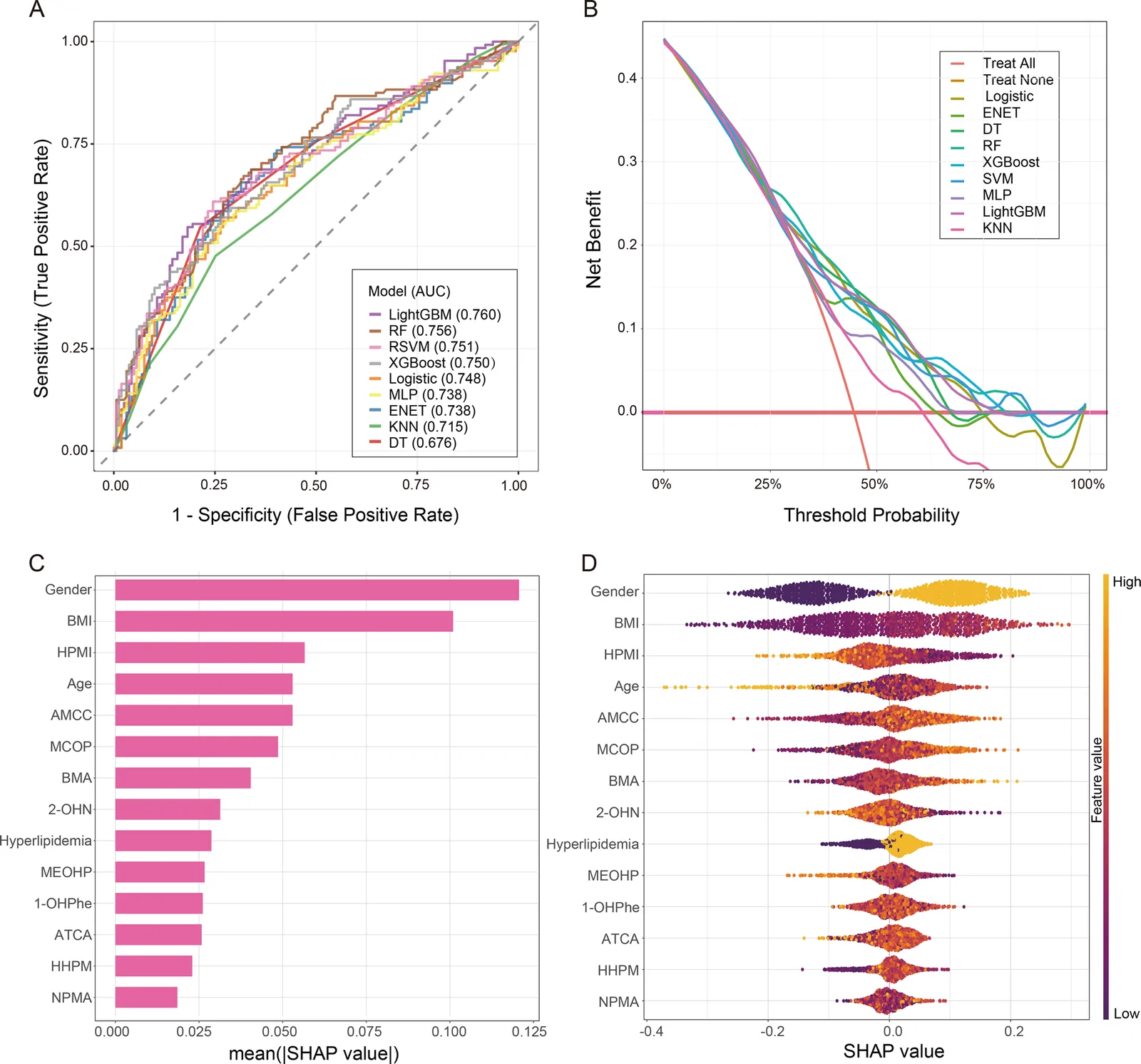
Machine learning model performance and feature importance for alcohol-related liver disease risk prediction. Performance evaluation and interpretability analysis of nine machine learning algorithms for predicting alcohol-related liver disease (ALD) risk in NHANES participants (2005–2006, 2011–2012, 2013–2014, 2015–2016). The dataset was randomly split 7:3 into training and test sets with stratified sampling. Feature selection was performed independently within each Fold of 5-Fold cross-validation using the intersection of features identified by both LASSO regression and Random Forest, yielding 14 consensus features: NPMA, BMA, AMCC, MCOP, HHPM, ATCA, 1-OHPhe, MEOHP, HPMI, 2-OHN, age, BMI, sex, and hyperlipidemia. Nine algorithms were evaluated: logistic regression (LR), elastic net (ENET), decision tree (DT), Random Forest (RF), extreme Gradient Boosting (XGBoost), support vector machine with radial basis function kernel (SVM-RBF), multi-layer perceptron (MLP), light Gradient Boosting machine (LightGBM), and K-Nearest neighbors (KNN). (**A**) Receiver operating characteristic (ROC) curves comparing discrimination performance of the nine algorithms on the held-out test set. The area under the curve (AUC) quantifies each model’s ability to distinguish between ALD cases and controls; curves closer to the upper-left corner indicate superior performance. (**B**) Decision curve analysis (DCA) evaluating the clinical utility of the nine models across a range of threshold probabilities. The y-axis represents net benefit, calculated as the difference between true positives and weighted false positives; curves above the “treat all” and “treat none” reference lines indicate clinical utility. (**C**) SHAP (SHapley Additive exPlanations) summary bar plot showing the mean absolute SHAP values for the 14 features in the final LightGBM model, ranked by overall importance. Higher values indicate greater contribution to model predictions. (**D**) SHAP beeswarm plot displaying the distribution of SHAP values for each feature across all test set participants. Each point represents an individual prediction; the x-axis shows SHAP values (contribution to log-odds of ALD risk), point position along the y-axis indicates feature ranking, and color represents feature value (red = high, blue = low). LightGBM achieved the highest cross-validation AUC (0.760, SD = 0.020) and was selected as the final model, with test set performance of AUC = 0.706 (95% CI: 0.690–0.723). All models incorporated NHANES sampling weights

LightGBM achieved the highest cross-validation AUC of 0.760 (SD = 0.020, range: 0.685–0.801) and was selected for comprehensive evaluation. On the held-out test set, LightGBM achieved an AUC of 0.706 (95% CI: 0.690–0.723) with balanced accuracy of 0.657 (detailed performance metrics in Table S8, Fig. 2A). The difference between cross-validation and test set AUC (ΔAUC = 0.054) indicated moderate overfitting despite methodological safeguards. The test set AUC of 0.706, while significantly above chance (*p* < 0.001), falls below the clinical utility threshold of 0.80. Calibration analysis showed good agreement between predicted and observed probabilities, and decision curve analysis revealed potential clinical utility pending external validation (Fig. 2B). These results represent internal validation within NHANES and require confirmation in independent cohorts before clinical application.

#### Feature contributions to ALD risk prediction

To interpret the predictive model and identify key contributing features, we applied SHAP (SHapley Additive exPlanations) analysis (Fig. 2C–D). The bar plot (Fig. 2C) ranked features by mean absolute SHAP value, with sex emerging as the most influential predictor, followed by hyperlipidemia and BMI among demographic factors. Among environmental exposures, HPMI, AMCC, and MCOP demonstrated the highest predictive importance.

The beeswarm plot (Fig. 2D) displayed SHAP value distributions for each feature, with dot position indicating contribution magnitude and direction, and color representing feature value (red for high, blue for low). Directional patterns revealed distinct effects across features. Male sex consistently showed large positive SHAP values, confirming markedly elevated ALD risk. HPMI predominantly exhibited negative SHAP values across its distribution, with higher exposure levels associated with negative contributions to ALD risk, suggesting potential protective effects or indication of effective detoxification capacity. In contrast, AMCC and MCOP displayed positive SHAP value distributions, with higher exposures consistently associated with increased ALD risk, confirming their hazardous potential. Hyperlipidemia and BMI showed expected positive associations.

Notably, several MDAs including MCOP demonstrated higher predictive importance than their statistical significance in regression models would suggest, indicating that machine learning captured non-linear relationships and interactions not detected by traditional parametric approaches.

In summary, among the evaluated MDA biomarkers, HPMI, AMCC, and MCOP emerged as the most critical features for ALD risk prediction in the machine learning framework, highlighting their potential clinical utility.

### Network toxicology analysis

**Fig. 3:**
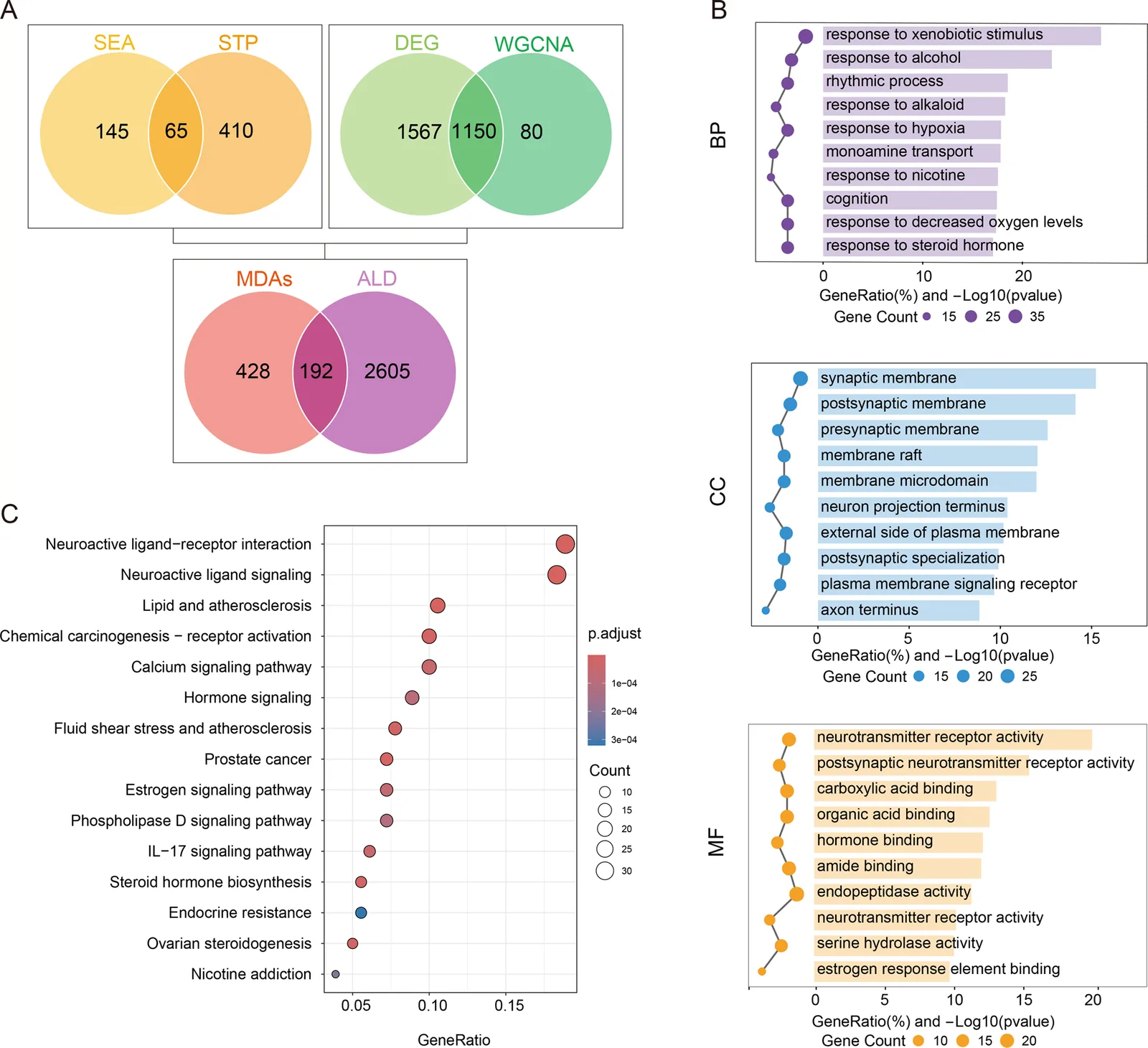
Network toxicology analysis identifying shared genes between metabolism-disrupting agent targets and alcohol-related liver disease-related pathways. Integrative network toxicology workflow to identify potential mechanistic mediators linking metabolism-disrupting agent (MDA) exposures to alcohol-related liver disease (ALD) pathogenesis. (**A**) Target identification and gene screening strategy. MDA protein targets were predicted using Swiss target prediction (STP, http://swisstargetprediction.ch/) and similarity ensemble approach (SEA, https://sea.bkslab.org/) databases, with the union of targets from both databases yielding the MDA target set. ALD-related genes were identified through two complementary approaches using gene expression Omnibus (GEO) dataset GSE28619 (liver tissue samples from 7 healthy controls and 15 ALD patients profiled on Affymetrix human genome U133 plus 2.0 arrays): (1) differential expression analysis using the limma package identified differentially expressed genes (DEGs) at thresholds of *p* < 0.05 and |log₂ fold-change| >1.2; (2) weighted gene co-expression network analysis (WGCNA) identified gene modules correlated with ALD phenotype, with soft-thresholding power (β) selected to achieve scale-free topology (R^2^ > 0.85), followed by hierarchical clustering with dynamic tree cutting to define modules. The union of DEGs and WGCNA module genes constituted the ALD-related gene set. The intersection of MDA targets and ALD-related genes yielded 192 shared genes representing candidate mechanistic mediators. (**B**) Gene ontology (GO) enrichment analysis of the 192 shared genes. Bubble plot displays the top 10 most significantly enriched terms in each GO category: biological process (BP), cellular Component (CC), and molecular function (MF). Bubble size represents the gene count (number of shared genes enriched in each term); bubble color indicates statistical significance (−log₁₀ adjusted *p*-value, Benjamini-Hochberg correction); the x-axis shows the gene ratio (proportion of shared genes annotated to each term). (**C**) Kyoto encyclopedia of genes and genomes (KEGG) pathway enrichment analysis of the 192 shared genes. Bubble plot displays the top 20 most significantly enriched KEGG pathways. Bubble size represents gene count; bubble color indicates −log₁₀ adjusted *p*-value (Benjamini-Hochberg correction); the x-axis shows the gene ratio. Enrichment analyses were performed using the clusterProfiler R package with significance threshold of adjusted *p* < 0.05. Key enriched pathways include “neuroactive ligand-receptor interaction” (52 genes, 27% of shared genes), “lipid and atherosclerosis” (33 genes), “IL-17 signaling pathway” (27 genes), and nuclear receptor signaling pathways (estrogen signaling, steroid hormone biosynthesis), suggesting MDAs and alcohol may converge on shared molecular mechanisms including nuclear receptor disruption, oxidative stress, and metabolic dysregulation

To generate mechanistic hypotheses linking observed MDA-ALD associations to biological pathways, we performed integrative network toxicology analysis. This approach identified 192 shared genes between computationally predicted MDA protein targets and ALD-related genes (DEGs plus WGCNA modules from GSE28619 dataset), representing potential mechanistic mediators (Fig. 3A, Figure S4).

GO enrichment analysis revealed functional characteristics directly relevant to ALD pathogenesis (Fig. 3B). In Biological Processes, the shared genes were significantly enriched in “cellular response to xenobiotic stimulus” (35 genes), “response to xenobiotic stimulus” (44 genes), “response to drug” (58 genes), and “response to ethanol” (31 genes; all adjusted *p* < 10^−19), directly linking MDA exposure to chemical detoxification and alcohol metabolism pathways. For Molecular Functions, top enrichments included “nuclear receptor activity” (15 genes), “ligand-activated transcription factor activity” (15 genes), “steroid hormone receptor activity” (11 genes), and “signaling receptor binding” (38 genes; all *p* < 10^−9), indicating disruption of nuclear receptor-mediated metabolic regulation. Cellular Component analysis revealed enrichment in “membrane raft” (29 genes), “receptor complex” (34 genes), “postsynaptic membrane” (28 genes), and “synaptic membrane” (29 genes; all *p* < 10^−11), suggesting MDAs concentrate in lipid-ordered membrane microdomains critical for signal transduction.

KEGG pathway enrichment identified key mechanistic pathways (Fig. 3C). “Neuroactive ligand-receptor interaction” was the most significantly enriched pathway (52 genes, 27% of shared genes; *p* = 4.32 × 10^−24), highlighting disruption of intercellular signaling as a primary mechanism. Pathways related to metabolic dysfunction included “Lipid and atherosclerosis” (33 genes), “IL-17 signaling pathway” (27 genes), and “Chemical carcinogenesis-receptor activation” (31 genes). Endocrine-related pathways included “Estrogen signaling pathway” (24 genes) and “Steroid hormone biosynthesis” (13 genes; all *p* < 10^−6). The convergence on nuclear receptor signaling pathways suggests MDAs and alcohol synergistically disrupt hepatic lipid homeostasis through shared molecular targets, particularly PPARα/γ, LXR, and FXR.

Collectively, these computational analyses generate the hypothesis that MDAs may function not primarily as direct hepatotoxins but rather as systemic signaling disruptors that may perturb neuro-endocrine-immune networks, potentially contributing to alcohol-induced liver injury through disruption of metabolic homeostasis. This mechanistic framework requires validation through targeted in vitro and in vivo experiments.

## Discussion

This study examined associations between environmental metabolism-disrupting agents (MDAs) and ALD risk using NHANES data. Several key findings emerged. Specific MDAs showed significant associations with ALD risk, including PFHxS, BP-3, and VOC metabolites (BMA, ATCA, AMCC). BMA showed the strongest association (Q4 OR: 1.56, 95% CI: 1.31–1.86), while MHBMA3 exhibited an inverse association, suggesting chemical-specific mechanisms. Dose-response patterns varied: PFHxS and BMA showed linear relationships without apparent thresholds, while ATCA and BP-3 exhibited non-linear patterns (inverted U-shaped and plateau curves, respectively), suggesting dose-dependent mechanisms. Machine learning approaches identified additional predictive MDAs (HPMI, MCOP, 2-OHP) not captured by traditional regression. Network toxicology analyses suggested that MDAs may act as signal disruptors in neuro-endocrine-immune pathways rather than direct hepatotoxins. Enrichment analyses highlighted neurotransmitter receptor activity and neuroactive ligand-receptor interactions. These findings are relevant given increasing alcohol consumption and widespread chemical exposures. Current ALD risk assessment does not incorporate environmental exposures, a gap this study addresses.

### Chemical-specific associations and dose-response Characteristics

We now examine individual MDAs demonstrating these associations. Among PFAS, perfluorohexanesulfonic acid (PFHxS) demonstrated a linear positive association with ALD risk across the full exposure range (P-overall < 0.001, P-non-linear = 0.052). PFHxS induces hepatotoxicity through PPAR pathway disruption and impaired lipid homeostasis [29, 30]. The absence of a threshold suggests risk even at low environmental exposures, particularly concerning given increasing PFHxS use as a PFAS replacement [31]. Mechanistically, PFAS downregulates lipid efflux and disrupts bile acid homeostasis, pathways implicated in ALD pathogenesis [32, 33]. The association was stronger among individuals with obesity and heavy drinking, supporting synergistic effects between environmental exposures, metabolic dysfunction, and alcohol [13].

Among VOC metabolites, benzylmercapturic acid (BMA) demonstrated the strongest association (Q4 OR: 1.56, 95% CI: 1.31–1.86) with a linear dose-response pattern across the entire exposure distribution. ATCA exhibited a pronounced inverted U-shaped dose-response pattern, with ALD risk increasing progressively at low-to-moderate exposure levels, reaching a peak at approximately the median-to-75th percentile range, and subsequently attenuating at higher concentrations. This non-monotonic pattern may reflect activation of compensatory hepatoprotective mechanisms at higher exposures, indicating that moderate-exposure populations may represent higher-priority intervention targets than those with the highest measured concentrations, and that non-monotonic models are more appropriate than linear approaches for ATCA risk assessment [34, 35].

Benzophenone-3 (BP-3) exhibited a non-linear pattern characterized by positive associations at low-to-moderate exposures that plateaued at higher concentrations. This plateau effect likely reflects receptor saturation (e.g., estrogenic receptor or aryl hydrocarbon receptor binding sites) at higher exposure levels, such that incremental increases above the plateau threshold do not proportionally increase hepatotoxic risk. This pattern indicates that linear extrapolation would overestimate risk at high exposures and that plateau-inclusive non-linear models are more appropriate for BP-3 risk characterization than conventional linear approaches.

MHBMA3, a 1,3-butadiene metabolite, demonstrated an inverse association (Q4 OR: 0.75, 95% CI: 0.59–0.94). This finding may reflect MHBMA3 as a biomarker of effective Phase II detoxification capacity rather than a causal protective agent. Individuals with higher urinary MHBMA3 concentrations may possess more robust glutathione conjugation systems. Such enhanced detoxification capacity could independently confer protection against alcohol-induced hepatic injury, potentially explaining the observed inverse association [36, 37]. However, this inverse association should be interpreted cautiously given the cross-sectional design, and validation in independent cohorts with mechanistic studies is needed to distinguish between biomarker effects and potential protective mechanisms [38].

The diversity of dose-response patterns—linear relationships without thresholds (PFHxS, BMA), inverted U-shaped non-monotonic patterns (ATCA), plateau effects (BP-3), and inverse associations (MHBMA3)—highlights chemical-specific risk profiles and challenges uniform linear assumptions [39, 40]. The absence of thresholds for PFHxS and BMA supports precautionary approaches, while, the inverted U-shaped pattern for ATCA suggests that moderate-exposure populations warrant prioritized intervention strategies [41].

### Machine learning enhancement of risk prediction

Machine learning approaches complemented and enhanced risk prediction by capturing non-linear relationships and high-order interactions among exposures that traditional regression cannot detect [42–44]. Traditional regression assumes linearity, which may not hold for complex MDA-ALD relationships involving multiple chemical exposures.

LightGBM achieved optimal performance (AUC: 0.760 in cross-validation), with improved predictive ability compared to traditional logistic regression (AUC: 0.748). SHAP analysis revealed HPMI, AMCC, and MCOP as key predictors. Importantly, MCOP showed substantial predictive importance despite non-significant regression results, suggesting effects mediated through exposure interactions that would have been missed by traditional methods alone [45]. Machine learning captured non-linear relationships and interactions not detected by linear models, identifying candidate predictive biomarkers (HPMI, MCOP) that emerged only through data-driven pattern recognition. Although machine learning methods are less directly interpretable than parametric models, SHAP analysis provided transparent feature contribution quantification, bridging this interpretability gap [46, 47]. While test set performance (AUC = 0.706) was lower than cross-validation estimates (ΔAUC = 0.054), indicating moderate overfitting that is not uncommon in complex models [48], we explicitly emphasize that independent external validation in demographically diverse cohorts is an absolute prerequisite to confirm model generalizability and safety before these algorithms can be considered for clinical application. Nevertheless, the complementary use of both approaches successfully revealed exposures and vulnerabilities not apparent in single-method analyses, demonstrating the added value of integrative analytical strategies.

### Mechanistic pathways underlying MDA-ALD interactions

To generate mechanistic hypotheses regarding biological mechanisms underlying observed associations, network toxicology analyses identified 192 shared genes between computationally predicted MDA targets and ALD-related pathways. Enrichment analyses suggested a potential pattern: MDAs may act primarily through neuro-endocrine signaling rather than direct hepatotoxicity, though this hypothesis requires experimental validation given typical false-positive rates of 30–50% in structure-based target predictions [49]. KEGG pathway analysis identified “neuroactive ligand-receptor interaction” as the most statistically enriched pathway (52 genes, *p* < 0.001), with GO terms highlighting neurotransmitter and neuropeptide receptor activities. While pathway enrichment reflects statistical over-representation and does not establish causality, this finding is consistent with the hypothesis that MDAs may function not only as direct hepatotoxins, but also as systemic modulators that could disrupt neuro-endocrine-immune signaling. At the cellular and systemic levels, endocrine-disrupting MDAs may perturb the hypothalamic-pituitary-adrenal (HPA) axis and autonomic tone. Because the liver is highly innervated, such systemic neuroendocrine perturbations can alter the local hepatic immune microenvironment, specifically by sensitizing resident macrophages (Kupffer cells) to alcohol-derived gut endotoxins. Combined with the enriched “IL-17 signaling pathway,” this neuro-immune crosstalk could precipitate an exaggerated inflammatory response, accelerating the transition from simple steatosis to severe steatohepatitis.

At the molecular level, nuclear receptors (PPARα/γ, PXR, CAR, FXR) regulate hepatic metabolism and detoxification, and represent candidate convergent targets for MDAs and alcohol based on computational predictions and prior experimental studies [50]. Direct experimental evidence confirms that specific PFAS activate PPARα [51, 52] and PXR [53, 54], supporting biological plausibility for our computational findings. If validated, their convergence could potentially create contradictory metabolic signals and additive impairments in lipid and bile acid homeostasis, favoring hepatic steatosis [13]. Furthermore, at the sub-cellular level, enriched biological processes included xenobiotic and alcohol responses, consistent with shared requirements for hepatic biotransformation and potential competitive inhibition [14]. Both MDAs (such as VOC metabolites) and alcohol rely heavily on shared hepatic enzymatic machinery. Their concurrent biotransformation can competitively inhibit or induce cytochrome P450 enzymes (particularly CYP2E1) while rapidly depleting intracellular glutathione antioxidant pools. This dual burden likely overwhelms phase II detoxification capacities, leading to massive oxidative stress. Indeed, both MDAs and alcohol are known to generate reactive oxygen species through mitochondrial dysfunction and enzymatic pathways [14, 16, 55], though whether these pathways synergistically interact in vivo requires direct experimental testing.

Collectively, these computational and enrichment analyses generate a mechanistic framework suggesting that MDA-ALD associations may involve interconnected pathways including nuclear receptor signaling, oxidative stress, neuro-immune crosstalk and metabolic dysregulation. If confirmed through experimental studies, this mechanistic overlap could help explain the observed associations and suggest that interventions targeting these pathways might potentially benefit individuals with combined exposures. However, given the cross-sectional nature of our epidemiological data and the computational basis of mechanistic inferences, these hypotheses require validation through: (1) prospective cohort studies establishing temporal relationships between MDA exposures and ALD incidence; (2) in vitro studies confirming MDA-receptor interactions and pathway perturbations; (3) animal models testing synergistic effects of combined MDA-alcohol exposures.

### Public Health and clinical translation

Translation of these findings into actionable strategies is warranted. Identified high-risk MDAs (BMA, ATCA, AMCC, PFHxS) warrant regulatory attention. Given increasing PFHxS use as a replacement for legacy long-chain PFAS, the absence of apparent thresholds for PFHxS and BMA challenges assumptions of safe exposure limits and supports precautionary regulatory approaches that minimize population-level exposure [41]. Regulatory frameworks should explicitly incorporate short-chain PFAS such as PFHxS into liver outcome assessments, given their increasing use as legacy PFAS substitutes and the hepatotoxic associations documented in this study [31, 56]. Source control measures targeting VOC-emitting products—including industrial emission reduction standards and improved indoor ventilation requirements—could meaningfully decrease population-level BMA and ATCA exposures [57, 58]. Non-linear patterns for ATCA and BP-3 highlight the need for flexible risk assessment approaches [59].

Clinically, incorporating environmental exposure histories into routine assessment could meaningfully enhance ALD risk stratification, particularly for patients with a history of heavy alcohol use. Heavy drinkers with known or suspected occupational and residential exposures to PFAS or VOC-emitting products represent a high-priority group for enhanced hepatic surveillance [14, 16]. Integrated assessment of both alcohol use and environmental exposures should be incorporated into occupational health programs and addiction treatment settings, with referral pathways to occupational medicine specialists established for high-risk patients [60]. However, clinical implementation faces barriers including testing costs, lack of standardized protocols, and limited provider training in environmental health [61]. Addressing these barriers will require development of cost-effective screening strategies, standardized clinical protocols for environmental exposure assessment, and integration of environmental health competencies into medical education and continuing professional development programs.

Future research should prioritize prospective studies to establish causality, intervention trials to test exposure reduction strategies, and mechanistic studies to identify therapeutic targets [62]. Addressing these dual threats requires coordinated efforts across regulatory, research, and clinical domains.

### Study limitations and future directions

This study has limitations. The cross-sectional design precludes causal inference [13]. Crucially, reverse causation cannot be excluded: because the liver governs xenobiotic biotransformation, pre-existing alcohol-induced hepatic impairment could alter MDA toxicokinetics and reduce their clearance, meaning elevated MDA levels might partially reflect disease consequence rather than the initial pathogenic driver. Longitudinal studies with repeated exposure assessments prior to disease onset are needed to establish temporality [38]. Single-spot urine measurements may not capture chronic exposures for rapidly metabolized chemicals, potentially causing exposure misclassification. ALD ascertainment may have favored advanced disease, potentially missing early-stage associations. Furthermore, because formal multiple testing correction was not applied to all initial analyses given the study’s exploratory nature, the possibility of false-positive findings among marginally significant associations cannot be completely ruled out. Residual confounding from unmeasured factors cannot be fully excluded. For instance, diet acts as a bidirectional confounder, serving simultaneously as a primary vector for MDA exposure and an independent driver of hepatic steatosis. Additionally, unmeasured functional polymorphisms in key metabolizing enzymes (e.g., ADH1B, ALDH2, CYP2E1, GSTM1/T1) govern individual susceptibility to both alcohol and MDA detoxification. External validation of machine learning models in independent cohorts is needed before clinical application [48]. Mechanistic inferences from network toxicology require experimental validation. Furthermore, future experimental investigations should prioritize assessing the synergistic hepatotoxic interactions between alcohol and specific high-risk MDAs identified in this study—particularly PFHxS, BMA, and ATCA—given their robust epidemiological associations and shared mechanistic convergence on lipid dysregulation, oxidative stress, and glutathione depletion.

## Conclusion

This study highlights the role of MDAs in ALD risk, demonstrating significant associations across several chemical classes (particularly PFHxS, BMA, and ATCA). Machine learning and network toxicology approaches offer new insights into MDA-ALD interactions and potential biomarkers (notably HPMI, AMCC, and MCOP). These findings emphasize the need for integrating environmental exposure assessments into ALD risk stratification and highlight the potential for regulatory action to mitigate these risks. Further research is needed to validate these findings and explore causal mechanisms underlying synergistic hepatotoxicity.

## Electronic supplementary material

Below is the link to the electronic supplementary material.

Supplementary Material 1

## Data Availability

The datasets analyzed during the current study are publicly available in the NHANES repository at https://www.cdc.gov/nchs/nhanes/index.htm. Gene expression data were obtained from the Gene Expression Omnibus (GEO) database under accession number GSE28619 (https://www.ncbi.nlm.nih.gov/geo/query/acc.cgi?acc=GSE28619). All R code and analytical scripts used for statistical analyses, machine learning model development, and network toxicology analyses are available from the corresponding authors upon reasonable request. Additional supplementary data supporting the findings of this study are included in the supplementary information files.
