# Supplementary material for: The hidden effects of metabolism-disrupting agents on alcohol-related liver disease risk: an integrated epidemiological, machine learning, and network toxicology Analysis
Source: J Transl Med. 2026 Jul 25;24:1328. doi: 10.1186/s12967-026-08673-2 (PMC13688738; doi:10.1186/s12967-026-08673-2)
Supplement: Supplementary file 1 [file 12967_2026_8673_MOESM1_ESM.docx]

**Table S1** Distribution Characteristics of 40 Metabolism-Disrupting Agents Measured in NHANES 2005-2006, 2011-2012, 2013-2014, and 2015-2016

**Table S2** Associations Between Metabolism-Disrupting Agents and Alcohol-Related Liver Disease Risk Across Exposure Quartiles

**Table S3** Age-Stratified Associations Between Metabolism-Disrupting Agents and Alcohol-Related Liver Disease Risk

**Table S4** Race/Ethnicity-Stratified Associations Between Metabolism-Disrupting Agents and Alcohol-Related Liver Disease Risk

**Table S5** BMI-Stratified Associations Between Metabolism-Disrupting Agents and Alcohol-Related Liver Disease Risk

**Table S6** Hyperlipidemia-Stratified Associations Between Metabolism-Disrupting Agents and Alcohol-Related Liver Disease Risk

**Table S7** Cross-Validation Performance Comparison of Machine Learning Algorithms for Alcohol-Related Liver Disease Risk Prediction

**Table S8** Performance Metrics of Machine Learning Algorithms on the Held-Out Test Set

**Figure S1** Flowchart of Participant Selection from NHANES 2005-2006, 2011-2012, 2013-2014, and 2015-2016

**Figure S2** Feature Selection Process Using LASSO Regression and Random Forest for Alcohol-Related Liver Disease Risk Prediction

**Figure S3 Dose-Response Relationships Between Additional Metabolism-Disrupting Agents and Alcohol-Related Liver Disease Risk**

**Figure S4** Identification of 192 Shared Genes Between Metabolism-Disrupting Agent Targets and Alcohol-Related Liver Disease-Related Genes

**Table S1** Distribution Characteristics of 40 Metabolism-Disrupting Agents Measured in NHANES 2005-2006, 2011-2012, 2013-2014, and 2015-2016

| MDAs | Abbreviation | N | GM | GSD | IQR |
| --- | --- | --- | --- | --- | --- |
| **PFAS** |  |  |  |  |  |
| Perfluorodecanoic acid | PFDeA | 4372 | 0.22 | 2.34 | 0.30 |
| Perfluorohexane sulfonic acid | PFHxS | 4372 | 1.30 | 2.64 | 1.60 |
| Perfluorononanoic acid | PFNA | 4372 | 0.81 | 2.10 | 0.80 |
| **PHEN** |  |  |  |  |  |
| Benzophenone-3 | BP-3 | 4313 | 18.04 | 8.94 | 62.50 |
| Bisphenol A | BPA | 4313 | 1.38 | 3.02 | 2.10 |
| Methyl paraben | MPB | 4313 | 54.88 | 5.91 | 197.10 |
| Propyl paraben | PPB | 4313 | 7.07 | 9.57 | 41.20 |
| 2,4-dichlorophenol | 2,4-DCP | 4313 | 0.81 | 4.34 | 1.40 |
| 2,5-dichlorophenol | 2,5-DCP | 4313 | 5.71 | 9.43 | 18.90 |
| **PHTH** |  |  |  |  |  |
| Mono(carboxyisononyl) phthalate | MCNP | 4313 | 2.21 | 3.07 | 3.30 |
| Mono(carboxyisoctyl) phthalate | MCOP | 4313 | 11.43 | 4.34 | 25.60 |
| Mono-2-ethyl-5-carboxypentyl phthalate | MECPP | 4313 | 13.51 | 3.33 | 19.90 |
| Mono-n-butyl phthalate | MnBP | 4313 | 10.66 | 3.59 | 18.80 |
| Mono-(3-carboxypropyl) phthalate | MCPP | 4313 | 1.86 | 3.77 | 3.30 |
| Mono-ethyl phthalate | MEP | 4313 | 55.71 | 5.08 | 141.58 |
| Mono-(2-ethyl-5-hydroxyhexyl) phthalate | MEHHP | 4313 | 8.76 | 3.54 | 14.10 |
| Mono-isobutyl phthalate | MiBP | 4313 | 6.89 | 3.23 | 11.10 |
| Mono-(2-ethyl-5-oxohexyl) phthalate | MEOHP | 4313 | 5.54 | 3.52 | 8.80 |
| Mono-benzyl phthalate | MBzP | 4313 | 4.51 | 3.79 | 9.33 |
| **PAH** |  |  |  |  |  |
| 1-Hydroxynapthalene | 1-OHN | 4925 | 2.66 | 5.25 | 7.89 |
| 2-Hydroxynapthalene | 2-OHN | 4925 | 5.52 | 3.33 | 11.02 |
| 3-Hydroxyfluorene | 3-OHF | 4925 | 0.13 | 4.51 | 0.38 |
| 2-Hydroxyfluorene | 2-OHF | 4925 | 0.31 | 3.80 | 0.68 |
| 1-Hydroxyphenanthrene | 1-OHPhe | 4925 | 0.13 | 2.65 | 0.17 |
| 1-Hydroxypyrene | 1-OHP | 4925 | 0.13 | 2.85 | 0.20 |
| **VOC Metabolites** |  |  |  |  |  |
| N-Acetyl-S-(2-carbamoylethyl)-L-cysteine | AAMA | 5140 | 61.42 | 2.92 | 94.90 |
| N-Acetyl-S-(N-methylcarbamoyl)-L-cysteine | AMCC | 5140 | 177.22 | 3.13 | 311.80 |
| 2-Aminothiazoline-4-carboxylic acid | ATCA | 5140 | 99.00 | 3.25 | 183.33 |
| N-Acetyl-S-(benzyl)-L-cysteine | BMA | 5140 | 7.09 | 2.95 | 10.07 |
| N-Acetyl-S-(n-propyl)-L-cysteine | NPMA | 5140 | 4.28 | 3.98 | 9.67 |
| N-Acetyl-S-(2-carboxyethyl)-L-cysteine | CEMA | 5140 | 113.73 | 2.94 | 171.10 |
| N-Acetyl-S-(2-cyanoethyl)-L-cysteine | CYMA | 5140 | 7.66 | 11.29 | 89.36 |
| N-Acetyl-S-(3,4-dihydroxybutyl)-L-cysteine | DHBMA | 5140 | 298.23 | 2.30 | 349.00 |
| N-Acetyl-S-(3-hydroxypropyl)-L-cysteine | HPMA | 5140 | 341.67 | 3.32 | 630.00 |
| N-Acetyl-S-(2-hydroxypropyl)-L-cysteine | HHPM | 5140 | 38.01 | 3.08 | 57.43 |
| 2-Methylhippuric acid | 2-MHA | 5140 | 40.29 | 3.40 | 83.00 |
| 3- and 4-Methylhippuric acid | 3/4-MHA | 5140 | 274.95 | 3.60 | 601.00 |
| N-Acetyl-S-(4-hydroxy-2-butenyl)-L-cysteine | MHBMA3 | 5140 | 8.30 | 3.85 | 18.90 |
| Phenylglyoxylic acid | PGA | 5140 | 176.85 | 3.09 | 271.00 |
| N-Acetyl-S-(3-hydroxypropyl-1-methyl)-L-cysteine | HPMI | 5140 | 356.56 | 3.40 | 674.00 |

Descriptive statistics for 40 metabolism-disrupting agents (MDAs) across five chemical classes measured in NHANES participants (2005-2006, 2011-2012, 2013-2014, 2015-2016). Sample sizes (N) vary by MDA due to differential availability across survey cycles and detection rates. Urinary MDAs (phenols, parabens, phthalate metabolites, PAH metabolites, and VOC metabolites) were measured in spot urine samples and creatinine-corrected (ng/mg creatinine); serum PFAS were measured in serum samples (ng/mL). Concentrations below the limit of detection (LOD) were imputed as LOD/2. GM (geometric mean) and GSD (geometric standard deviation) were calculated from log-transformed concentrations to account for right-skewed distributions typical of environmental biomarker data. IQR (interquartile range) represents the difference between the 75th and 25th percentiles of the original concentration distribution. All measurements were performed at the CDC Division of Laboratory Sciences using liquid chromatography-tandem mass spectrometry (LC-MS/MS) with class-specific sample preparation protocols detailed in NHANES laboratory documentation (available at [www.cdc.gov/nchs/nhanes](http://www.cdc.gov/nchs/nhanes) ).Sample sizes vary due to differential availability of measurements across NHANES survey cycles and differences in detection rates for individual analytes.

Abbreviations: GM, geometric mean; GSD, geometric standard deviation; IQR, interquartile range; PFAS, per- and polyfluoroalkyl substances; PHEN, phenols and parabens; PHTH, phthalate metabolites; PAH, polycyclic aromatic hydrocarbon metabolites; mVOC, volatile organic compound metabolites. Individual MDA abbreviations and full chemical names are listed in the table.

**Table S2** Associations Between Metabolism-Disrupting Agents and Alcohol-Related Liver Disease Risk Across Exposure Quartiles

|  |  | Model 1  (OR 95%CI) | P | Model 2  (OR 95%CI) | P | Model 3  (OR 95%CI) | P |
| --- | --- | --- | --- | --- | --- | --- | --- |
| **PFASs (Log ng/mL)** | | | |  |  |  |  |
| PFDeA |  |  |  |  |  |  |  |
|  | Q1 | Ref. |  | Ref. |  | Ref. |  |
|  | Q2 | 1.02(0.87,1.20) | 0.78 | 0.94(0.80,1.12) | 0.51 | 0.99(0.83,1.19) | 0.93 |
|  | Q3 | 1.09(0.93,1.29) | 0.29 | 1.01(0.85,1.20) | 0.95 | 1.01(0.84,1.22) | 0.89 |
|  | Q4 | 0.90(0.75,1.08) | 0.29 | 0.89(0.74,1.08) | 0.25 | 0.99(0.80,1.21) | 0.90 |
| **Phenols and Parabens (Log ng/mg CR)** | | | |  |  |  |  |
| BPA |  |  |  |  |  |  |  |
|  | Q1 | Ref. |  | Ref. |  | Ref. |  |
|  | Q2 | 1.03(0.87,1.22) | 0.73 | 1.04(0.87,1.24) | 0.69 | 1.06(0.87,1.27) | 0.58 |
|  | Q3 | 1.11(0.94,1.31) | 0.24 | 1.10(0.91,1.31) | 0.32 | 1.02(0.85,1.24) | 0.82 |
|  | Q4 | 0.99(0.84,1.18) | 0.93 | 1.04(0.87,1.25) | 0.68 | 1.00(0.83,1.22) | 0.97 |
| 2,5-DCP |  |  |  |  |  |  |  |
|  | Q1 | Ref. |  | Ref. |  | Ref. |  |
|  | Q2 | 1.10(0.92,1.30) | 0.29 | 1.11(0.92,1.32) | 0.27 | 1.04(0.86,1.25) | 0.7 |
|  | Q3 | 1.12(0.95,1.33) | 0.17 | 1.15(0.96,1.38) | 0.14 | 1.14(0.94,1.39) | 0.18 |
|  | Q4 | 0.87(0.73,1.03) | 0.11 | 0.99(0.82,1.20) | 0.92 | 1.02(0.84,1.25) | 0.83 |
| **Phthalate Metabolites (Log ng/mg CR)** | | | |  |  |  |  |
| MCNP |  |  |  |  |  |  |  |
|  | Q1 | Ref. |  | Ref. |  | Ref. |  |
|  | Q2 | 1.26(1.07,1.50) | 0.01 | 1.26(1.05,1.51) | 0.01 | 1.20(0.99,1.45) | 0.06 |
|  | Q3 | 1.25(1.05,1.48) | 0.01 | 1.28(1.07,1.54) | 0.01 | 1.20(0.99,1.45) | 0.06 |
|  | Q4 | 1.19(1.00,1.41) | 0.05 | 1.21(1.01,1.45) | 0.04 | 1.13(0.93,1.37) | 0.21 |
| MCOP |  |  |  |  |  |  |  |
|  | Q1 | Ref. |  | Ref. |  | Ref. |  |
|  | Q2 | 1.04(0.87,1.23) | 0.68 | 1.08(0.90,1.29) | 0.43 | 1.01(0.83,1.22) | 0.95 |
|  | Q3 | 1.06(0.89,1.26) | 0.50 | 1.13(0.94,1.35) | 0.19 | 1.05(0.87,1.26) | 0.64 |
|  | Q4 | 1.14(0.96,1.35) | 0.14 | 1.24(1.04,1.49) | 0.02 | 1.08(0.89,1.31) | 0.42 |
| MECPP |  |  |  |  |  |  |  |
|  | Q1 | Ref. |  | Ref. |  | Ref. |  |
|  | Q2 | 1.02(0.86,1.21) | 0.78 | 1.01(0.84,1.21) | 0.93 | 0.98(0.81,1.18) | 0.83 |
|  | Q3 | 1.00(0.84,1.18) | 0.98 | 1.00(0.84,1.20) | 1.00 | 0.95(0.79,1.15) | 0.62 |
|  | Q4 | 0.93(0.79,1.11) | 0.42 | 1.01(0.84,1.21) | 0.94 | 0.97(0.80,1.17) | 0.75 |
| MnBP |  |  |  |  |  |  |  |
|  | Q1 | Ref. |  | Ref. |  | Ref. |  |
|  | Q2 | 0.91(0.77,1.08) | 0.28 | 0.91(0.76,1.09) | 0.31 | 0.86(0.72,1.04) | 0.13 |
|  | Q3 | 0.92(0.78,1.09) | 0.32 | 0.94(0.78,1.12) | 0.46 | 0.87(0.72,1.05) | 0.16 |
|  | Q4 | 0.74(0.62,0.88) | <0.001 | 0.87(0.73,1.04) | 0.14 | 0.87(0.72,1.05) | 0.15 |
| MCPP |  |  |  |  |  |  |  |
|  | Q1 | Ref. |  | Ref. |  | Ref. |  |
|  | Q2 | 1.05(0.89,1.25) | 0.56 | 1.07(0.89,1.28) | 0.48 | 1.03(0.85,1.24) | 0.78 |
|  | Q3 | 1.00(0.84,1.18) | 0.99 | 0.97(0.81,1.17) | 0.77 | 0.94(0.78,1.14) | 0.52 |
|  | Q4 | 1.00(0.84,1.18) | 1.00 | 1.02(0.85,1.22) | 0.84 | 0.97(0.80,1.18) | 0.77 |
| MEHHP |  |  |  |  |  |  |  |
|  | Q1 | Ref. |  | Ref. |  | Ref. |  |
|  | Q2 | 1.10(0.93,1.30) | 0.27 | 1.05(0.88,1.25) | 0.61 | 1.01(0.84,1.22) | 0.90 |
|  | Q3 | 1.01(0.85,1.19) | 0.95 | 1.00(0.83,1.19) | 0.98 | 0.89(0.74,1.08) | 0.23 |
|  | Q4 | 0.99(0.83,1.17) | 0.88 | 1.05(0.87,1.26) | 0.61 | 1.01(0.83,1.22) | 0.92 |
| MBzP |  |  |  |  |  |  |  |
|  | Q1 | Ref. |  | Ref. |  | Ref. |  |
|  | Q2 | 1.09(0.92,1.29) | 0.30 | 1.09(0.91,1.30) | 0.34 | 1.07(0.89,1.29) | 0.48 |
|  | Q3 | 0.94(0.79,1.11) | 0.46 | 0.98(0.82,1.17) | 0.79 | 0.96(0.79,1.16) | 0.65 |
|  | Q4 | 0.85(0.72,1.01) | 0.06 | 0.90(0.75,1.08) | 0.25 | 0.88(0.73,1.08) | 0.22 |
| **PAH Metabolites (Log ng/mg CR)** | | | |  |  |  |  |
| 1-OHN |  |  |  |  |  |  |  |
|  | Q1 | Ref. |  | Ref. |  | Ref. |  |
|  | Q2 | 1.11(0.95,1.30) | 0.19 | 1.11(0.94,1.32) | 0.20 | 1.08(0.91,1.29) | 0.37 |
|  | Q3 | 1.12(0.96,1.31) | 0.15 | 1.09(0.92,1.29) | 0.30 | 1.16(0.96,1.40) | 0.12 |
|  | Q4 | 1.01(0.86,1.18) | 0.89 | 0.95(0.81,1.13) | 0.59 | 1.01(0.81,1.25) | 0.94 |
| 2-OHN |  |  |  |  |  |  |  |
|  | Q1 | Ref. |  | Ref. |  | Ref. |  |
|  | Q2 | 1.10(0.94,1.29) | 0.23 | 1.06(0.90,1.26) | 0.49 | 1.02(0.85,1.22) | 0.81 |
|  | Q3 | 1.15(0.98,1.35) | 0.09 | 1.12(0.95,1.33) | 0.18 | 1.08(0.89,1.31) | 0.42 |
|  | Q4 | 1.02(0.87,1.20) | 0.76 | 1.02(0.86,1.21) | 0.80 | 0.99(0.81,1.22) | 0.94 |
| 3-OHF |  |  |  |  |  |  |  |
|  | Q1 | Ref. |  | Ref. |  | Ref. |  |
|  | Q2 | 1.23(1.05,1.44) | 0.01 | 1.17(0.99,1.38) | 0.07 | 1.13(0.95,1.35) | 0.17 |
|  | Q3 | 1.15(0.99,1.35) | 0.08 | 1.05(0.89,1.24) | 0.58 | 1.07(0.89,1.29) | 0.48 |
|  | Q4 | 1.16(0.99,1.36) | 0.06 | 0.99(0.84,1.18) | 0.93 | 1.07(0.84,1.35) | 0.60 |
| 2-OHF |  |  |  |  |  |  |  |
|  | Q1 | Ref. |  | Ref. |  | Ref. |  |
|  | Q2 | 1.24(1.06,1.46) | 0.01 | 1.21(1.02,1.43) | 0.03 | 1.17(0.98,1.39) | 0.09 |
|  | Q3 | 1.10(0.94,1.28) | 0.26 | 1.00(0.84,1.18) | 0.98 | 1.00(0.83,1.21) | 0.99 |
|  | Q4 | 1.19(1.01,1.39) | 0.03 | 1.04(0.88,1.24) | 0.63 | 1.04(0.82,1.30) | 0.76 |
| 1-OHP |  |  |  |  |  |  |  |
|  | Q1 | Ref. |  | Ref. |  | Ref. |  |
|  | Q2 | 1.05(0.90,1.24) | 0.51 | 1.08(0.91,1.27) | 0.38 | 1.15(0.96,1.37) | 0.12 |
|  | Q3 | 1.02(0.87,1.20) | 0.80 | 1.05(0.89,1.24) | 0.56 | 1.13(0.94,1.35) | 0.21 |
|  | Q4 | 1.08(0.92,1.27) | 0.32 | 1.08(0.91,1.28) | 0.39 | 1.21(0.99,1.48) | 0.06 |
| **VOC Metabolites (Log ng/mg CR)** | | | |  |  |  |  |
| AAMA |  |  |  |  |  |  |  |
|  | Q1 | Ref. |  | Ref. |  | Ref. |  |
|  | Q2 | 1.14(0.98,1.34) | 0.09 | 1.05(0.89,1.24) | 0.54 | 1.04(0.88,1.24) | 0.63 |
|  | Q3 | 1.13(0.97,1.32) | 0.13 | 1.00(0.84,1.17) | 0.96 | 0.99(0.83,1.19) | 0.95 |
|  | Q4 | 1.28(1.09,1.49) | 0.002 | 1.08(0.91,1.28) | 0.37 | 1.16(0.95,1.41) | 0.15 |
| CEMA |  |  |  |  |  |  |  |
|  | Q1 | Ref. |  | Ref. |  | Ref. |  |
|  | Q2 | 1.10(0.94,1.29) | 0.22 | 1.05(0.89,1.23) | 0.57 | 1.02(0.86,1.22) | 0.79 |
|  | Q3 | 1.20(1.02,1.40) | 0.02 | 1.12(0.95,1.32) | 0.18 | 1.04(0.87,1.25) | 0.63 |
|  | Q4 | 1.13(0.97,1.32) | 0.12 | 1.10(0.94,1.30) | 0.24 | 0.99(0.81,1.20) | 0.92 |
| DHBMA |  |  |  |  |  |  |  |
|  | Q1 | Ref. |  | Ref. |  | Ref. |  |
|  | Q2 | 1.09(0.93,1.27) | 0.29 | 1.05(0.90,1.24) | 0.53 | 1.04(0.88,1.24) | 0.64 |
|  | Q3 | 1.19(1.02,1.38) | 0.03 | 1.14(0.97,1.34) | 0.13 | 1.07(0.90,1.27) | 0.43 |
|  | Q4 | 1.15(0.98,1.34) | 0.08 | 1.14(0.97,1.34) | 0.12 | 1.09(0.91,1.31) | 0.33 |
| HPMA |  |  |  |  |  |  |  |
|  | Q1 | Ref. |  | Ref. |  | Ref. |  |
|  | Q2 | 1.21(1.04,1.41) | 0.02 | 1.09(0.93,1.29) | 0.29 | 1.01(0.85,1.21) | 0.89 |
|  | Q3 | 1.14(0.97,1.33) | 0.11 | 0.99(0.84,1.17) | 0.9 | 0.90(0.75,1.08) | 0.27 |
|  | Q4 | 1.04(0.89,1.22) | 0.58 | 0.93(0.79,1.09) | 0.38 | 0.89(0.72,1.09) | 0.26 |
| HHPM |  |  |  |  |  |  |  |
|  | Q1 | Ref. |  | Ref. |  | Ref. |  |
|  | Q2 | 1.05(0.90,1.23) | 0.51 | 0.98(0.83,1.16) | 0.82 | 0.99(0.83,1.18) | 0.90 |
|  | Q3 | 1.16(0.99,1.35) | 0.06 | 1.07(0.91,1.26) | 0.40 | 1.09(0.92,1.30) | 0.33 |
|  | Q4 | 1.09(0.93,1.27) | 0.27 | 1.04(0.88,1.22) | 0.65 | 1.05(0.88,1.27) | 0.58 |
| 2-MHA |  |  |  |  |  |  |  |
|  | Q1 | Ref. |  | Ref. |  | Ref. |  |
|  | Q2 | 1.12(0.96,1.31) | 0.16 | 1.04(0.88,1.22) | 0.67 | 1.02(0.86,1.21) | 0.84 |
|  | Q3 | 1.13(0.97,1.32) | 0.12 | 1.01(0.86,1.19) | 0.90 | 1.09(0.91,1.31) | 0.35 |
|  | Q4 | 1.13(0.97,1.32) | 0.11 | 1.00(0.85,1.18) | 1.00 | 1.09(0.89,1.33) | 0.40 |
| PGA |  |  |  |  |  |  |  |
|  | Q1 | Ref. |  | Ref. |  | Ref. |  |
|  | Q2 | 1.12(0.96,1.30) | 0.17 | 1.09(0.92,1.28) | 0.32 | 1.05(0.88,1.24) | 0.61 |
|  | Q3 | 1.10(0.94,1.29) | 0.22 | 1.06(0.90,1.24) | 0.50 | 1.03(0.86,1.23) | 0.74 |
|  | Q4 | 1.23(1.05,1.43) | 0.01 | 1.18(1.00,1.39) | 0.05 | 1.20(0.99,1.44) | 0.06 |
| CYMA |  |  |  |  |  |  |  |
|  | Q1 | Ref. |  |  |  |  |  |
|  | Q2 | 1.14(0.98,1.33) | 0.10 | 1.11(0.94,1.31) | 0.20 | 1.08(0.91,1.28) | 0.38 |
|  | Q3 | 1.16(1.00,1.36) | 0.06 | 1.04(0.88,1.22) | 0.66 | 1.08(0.88,1.32) | 0.45 |
|  | Q4 | 1.23(1.05,1.43) | 0.01 | 1.08(0.91,1.27) | 0.38 | 1.34(1.03,1.74) | 0.03 |
| HPMI |  |  |  |  |  |  |  |
|  | Q1 | Ref. |  | Ref. |  | Ref. |  |
|  | Q2 | 1.06(0.91,1.23) | 0.48 | 1.00(0.85,1.18) | 0.97 | 0.96(0.81,1.14) | 0.63 |
|  | Q3 | 0.99(0.85,1.16) | 0.91 | 0.94(0.80,1.11) | 0.46 | 0.85(0.71,1.02) | 0.08 |
|  | Q4 | 0.93(0.80,1.09) | 0.36 | 0.85(0.72,1.00) | 0.05 | 0.80(0.65,1.00) | 0.05 |

Odds ratios (OR) and 95% confidence intervals (CI) for associations between metabolism-disrupting agent (MDA) exposures and alcohol-related liver disease (ALD) risk in 13,472 NHANES participants (2005-2006, 2011-2012, 2013-2014, 2015-2016). This table complements Table 2 by presenting complete results for additional MDAs. MDA concentrations were log₁₀-transformed, winsorized at the 1st and 99th percentiles, and categorized into exposure quartiles (Q1-Q4), with the lowest quartile (Q1) serving as the reference group. Three logistic regression models were constructed: Model 1 (unadjusted); Model 2 (adjusted for age, sex, and race/ethnicity); Model 3 (fully adjusted for age, sex, race/ethnicity, body mass index [BMI], family income-to-poverty ratio [PIR], education level, smoking status, hypertension, and hyperlipidemia). All models incorporated NHANES sampling weights to account for the complex survey design and generate nationally representative estimates. Urinary MDA concentrations were creatinine-corrected (ng/mg creatinine [CR]); serum PFAS concentrations are reported in ng/mL. Statistical significance was set at two-sided P < 0.05.

**Table S3** Age-Stratified Associations Between Metabolism-Disrupting Agents and Alcohol-Related Liver Disease Risk

| Age | 20-39 |  | 40-59 |  | >=60 |  |  |
| --- | --- | --- | --- | --- | --- | --- | --- |
| Variable | OR(CI) | P value | OR(CI) | P value | OR(CI) | P value | P-int |
| **PFAS** |  |  |  |  |  |  |  |
| PFeDA | 1.022 (0.92-1.136) | 0.68 | 0.93 (0.841-1.029) | 0.16 | 1.071 (0.961-1.193) | 0.21 | 0.16 |
| PFHxS | 1.496 (1.355-1.655) | **0.00** | 1.479 (1.322-1.658) | **0.00** | 1.342 (1.177-1.535) | **0.00** | 0.41 |
| PFNA | 1.215 (1.098-1.347) | **0.00** | 1.122 (1.009-1.249) | **0.03** | 1.116 (0.998-1.25) | 0.06 | 0.45 |
| **PHEN** |  |  |  |  |  |  |  |
| BP3 | 0.909 (0.816-1.012) | 0.08 | 0.946 (0.853-1.048) | 0.29 | 1.252 (1.125-1.395) | **0.00** | **0.00** |
| BPA | 0.968 (0.876-1.069) | 0.52 | 0.997 (0.895-1.11) | 0.96 | 1.074 (0.961-1.2) | 0.21 | 0.38 |
| MBP | 0.764 (0.691-0.845) | **0.00** | 0.763 (0.686-0.849) | **0.00** | 0.908 (0.811-1.016) | 0.09 | **0.04** |
| PPB | 0.696 (0.626-0.772) | **0.00** | 0.772 (0.693-0.858) | **0.00** | 0.947 (0.85-1.056) | 0.33 | **0.00** |
| 24DCP | 0.907 (0.821-1.001) | 0.05 | 0.936 (0.841-1.041) | 0.22 | 0.937 (0.839-1.046) | 0.25 | 0.88 |
| 25DCP | 0.994 (0.902-1.094) | 0.90 | 0.924 (0.83-1.029) | 0.15 | 0.912 (0.814-1.021) | 0.11 | 0.45 |
| **PHTH** |  |  |  |  |  |  |  |
| MCNP | 1.006 (0.913-1.108) | 0.91 | 1.029 (0.926-1.143) | 0.60 | 1.192 (1.061-1.341) | **0.00** | 0.07 |
| MCOP | 1.04 (0.947-1.143) | 0.41 | 0.989 (0.889-1.1) | 0.84 | 1.115 (0.988-1.258) | 0.08 | 0.35 |
| MECPP | 0.944 (0.859-1.038) | 0.24 | 0.991 (0.893-1.099) | 0.86 | 1.062 (0.942-1.198) | 0.32 | 0.32 |
| MnBP | 0.895 (0.814-0.983) | **0.02** | 0.891 (0.801-0.99) | **0.03** | 1.022 (0.908-1.151) | 0.72 | 0.16 |
| MCPP | 1.017 (0.925-1.118) | 0.73 | 0.968 (0.87-1.078) | 0.56 | 1.031 (0.917-1.159) | 0.61 | 0.70 |
| MEP | 0.957 (0.866-1.057) | 0.39 | 0.958 (0.862-1.064) | 0.42 | 1.068 (0.958-1.192) | 0.24 | 0.26 |
| MEHHP | 0.966 (0.878-1.061) | 0.47 | 0.983 (0.886-1.09) | 0.75 | 1.084 (0.961-1.223) | 0.19 | 0.31 |
| MiBP | 0.881 (0.798-0.972) | **0.01** | 0.93 (0.835-1.035) | 0.18 | 1.049 (0.937-1.174) | 0.41 | 0.07 |
| MEOHP | 0.922 (0.838-1.014) | 0.09 | 0.926 (0.834-1.027) | 0.15 | 1.018 (0.904-1.148) | 0.76 | 0.39 |
| MBzP | 0.867 (0.787-0.954) | **0.00** | 1.015 (0.911-1.131) | 0.79 | 0.922 (0.821-1.036) | 0.17 | 0.10 |
| **PAH** |  |  |  |  |  |  |  |
| 1.OHN | 0.936 (0.853-1.026) | 0.16 | 0.958 (0.872-1.054) | 0.38 | 1.062 (0.954-1.183) | 0.27 | 0.19 |
| 2.OHN | 0.964 (0.878-1.058) | 0.44 | 0.997 (0.902-1.101) | 0.95 | 1.029 (0.925-1.143) | 0.60 | 0.66 |
| 2.OHF | 0.944 (0.86-1.037) | 0.23 | 1.033 (0.94-1.135) | 0.50 | 1.078 (0.965-1.205) | 0.18 | 0.17 |
| 3.OHF | 0.931 (0.848-1.022) | 0.13 | 1.04 (0.946-1.143) | 0.42 | 1.109 (0.992-1.24) | 0.07 | 0.05 |
| 1.OHPHE | 0.917 (0.836-1.005) | 0.06 | 1.041 (0.945-1.146) | 0.42 | 1.205 (1.08-1.345) | **0.00** | **0.00** |
| 1.OHP | 0.887 (0.808-0.974) | **0.01** | 1.003 (0.909-1.108) | 0.95 | 1.175 (1.051-1.314) | **0.01** | **0.00** |
| **VOC Metabolites** | |  |  |  |  |  |  |
| AAMA | 1.012 (0.928-1.103) | 0.79 | 1.036 (0.94-1.141) | 0.48 | 1.174 (1.046-1.319) | **0.01** | 0.11 |
| AMCC | 1.03 (0.942-1.125) | 0.52 | 1.058 (0.965-1.16) | 0.23 | 1.073 (0.96-1.199) | 0.21 | 0.84 |
| ATCA | 0.802 (0.733-0.877) | **0.00** | 0.857 (0.777-0.944) | **0.00** | 1.058 (0.953-1.176) | 0.29 | **0.00** |
| BMA | 1.002 (0.917-1.094) | 0.97 | 1.066 (0.97-1.171) | 0.19 | 1.258 (1.129-1.404) | **0.00** | **0.01** |
| NPMA | 0.951 (0.87-1.039) | 0.27 | 0.908 (0.826-0.998) | **0.05** | 1.298 (1.163-1.45) | **0.00** | **0.00** |
| CEMA | 1.083 (0.989-1.186) | 0.08 | 0.975 (0.89-1.069) | 0.59 | 1.094 (0.983-1.219) | 0.10 | 0.18 |
| CYMA | 1.064 (0.973-1.163) | 0.17 | 1.017 (0.928-1.116) | 0.72 | 1.018 (0.909-1.14) | 0.76 | 0.75 |
| DHBMA | 1.023 (0.936-1.119) | 0.61 | 1.035 (0.943-1.136) | 0.46 | 1.099 (0.987-1.225) | 0.09 | 0.58 |
| HPMA | 0.94 (0.856-1.032) | 0.19 | 0.931 (0.85-1.02) | 0.13 | 1.095 (0.984-1.218) | 0.10 | **0.05** |
| HHPM | 0.984 (0.897-1.079) | 0.74 | 0.942 (0.857-1.035) | 0.21 | 1.145 (1.032-1.27) | **0.01** | **0.02** |
| 2.MHA | 1.012 (0.923-1.109) | 0.80 | 1.017 (0.927-1.117) | 0.72 | 1.128 (1.015-1.255) | **0.03** | 0.25 |
| 3.4.MHA | 1.045 (0.955-1.143) | 0.34 | 1.057 (0.964-1.159) | 0.24 | 1.124 (1.007-1.256) | **0.04** | 0.57 |
| MHBMA3 | 0.954 (0.872-1.043) | 0.30 | 0.918 (0.838-1.006) | 0.07 | 0.986 (0.881-1.102) | 0.80 | 0.62 |
| PGA | 0.963 (0.881-1.053) | 0.41 | 1.017 (0.926-1.117) | 0.72 | 1.015 (0.912-1.131) | 0.79 | 0.65 |
| HPMI | 0.949 (0.866-1.039) | 0.26 | 0.935 (0.855-1.023) | 0.14 | 0.999 (0.896-1.115) | 0.99 | 0.64 |

Odds ratios (OR) and 95% confidence intervals (CI) for associations between metabolism-disrupting agent (MDA) exposures and alcohol-related liver disease (ALD) risk, stratified by age group. MDA concentrations were log₁₀-transformed, winsorized at the 1st and 99th percentiles, and standardized to Z-scores (mean=0, SD=1); ORs represent the change in ALD risk per 1-standard deviation increase in MDA concentration. Logistic regression models were adjusted for sex, race/ethnicity, body mass index (BMI), family income-to-poverty ratio (PIR), education level, smoking status, hypertension, and hyperlipidemia. All models incorporated NHANES sampling weights to account for the complex survey design. P-int represents the P-value for the multiplicative interaction term (MDA × age) derived from Wald tests of the interaction coefficient in logistic regression models. Urinary MDA concentrations were creatinine-corrected (ng/mg creatinine [CR]); serum PFAS concentrations are reported in ng/mL. Bold P-values indicate statistical significance (P < 0.05, two-sided).

**Table S4** Race/Ethnicity-Stratified Associations Between Metabolism-Disrupting Agents and Alcohol-Related Liver Disease Risk

| Race | Non-Hispanic White | | Mexican American | | Non-Hispanic Black | | Other Race | |  |
| --- | --- | --- | --- | --- | --- | --- | --- | --- | --- |
| Variable | OR(CI) | P value | OR(CI) | P value | OR(CI) | P value | OR(CI) | P value | P-int |
| **PFAS** |  |  |  |  |  |  |  |  |  |
| PFeDA | 1.213 (1.013-1.456) | **0.04** | 1.076 (0.947-1.221) | 0.26 | 1.05 (0.954-1.157) | 0.32 | 0.849 (0.754-0.955) | **0.01** | **0.00** |
| PFHxS | 1.63 (1.374-1.947) | **0.00** | 1.319 (1.159-1.506) | **0.00** | 1.304 (1.181-1.442) | **0.00** | 1.434 (1.252-1.649) | **0.00** | 0.13 |
| PFNA | 1.422 (1.198-1.699) | **0.00** | 1.14 (1.005-1.296) | **0.04** | 1.151 (1.048-1.266) | **0.00** | 1.038 (0.912-1.181) | 0.57 | **0.04** |
| **PHEN** |  |  |  |  |  |  |  |  |  |
| BP3 | 0.954 (0.803-1.132) | 0.59 | 0.948 (0.823-1.09) | 0.45 | 1.084 (0.991-1.186) | 0.08 | 0.958 (0.841-1.091) | 0.52 | 0.24 |
| BPA | 0.983 (0.837-1.154) | 0.84 | 0.932 (0.818-1.062) | 0.29 | 1.093 (0.995-1.201) | 0.06 | 1.043 (0.917-1.185) | 0.52 | 0.25 |
| MBP | 0.885 (0.747-1.047) | 0.16 | 0.904 (0.791-1.034) | 0.14 | 0.822 (0.746-0.904) | **0.00** | 0.775 (0.678-0.884) | **0.00** | 0.37 |
| PPB | 0.86 (0.733-1.008) | 0.06 | 0.866 (0.751-0.998) | **0.05** | 0.826 (0.752-0.907) | **0.00** | 0.77 (0.675-0.877) | **0.00** | 0.62 |
| 24DCP | 1.01 (0.87-1.174) | 0.90 | 0.932 (0.824-1.053) | 0.26 | 0.995 (0.892-1.109) | 0.93 | 0.9 (0.791-1.024) | 0.11 | 0.57 |
| 25DCP | 0.995 (0.853-1.161) | 0.95 | 0.961 (0.841-1.097) | 0.56 | 1.022 (0.915-1.142) | 0.70 | 0.989 (0.874-1.12) | 0.87 | 0.92 |
| **PHTH** |  |  |  |  |  |  |  |  |  |
| MCNP | 1.19 (0.998-1.422) | 0.05 | 0.919 (0.807-1.045) | 0.20 | 1.069 (0.973-1.175) | 0.17 | 1.179 (1.043-1.334) | **0.01** | **0.03** |
| MCOP | 1.066 (0.897-1.268) | 0.47 | 0.943 (0.829-1.073) | 0.38 | 1.048 (0.955-1.151) | 0.32 | 1.208 (1.066-1.371) | **0.00** | 0.06 |
| MECPP | 0.909 (0.771-1.07) | 0.25 | 0.945 (0.83-1.076) | 0.39 | 1.09 (0.993-1.197) | 0.07 | 0.932 (0.818-1.062) | 0.29 | 0.09 |
| MnBP | 0.94 (0.796-1.108) | 0.46 | 0.932 (0.816-1.064) | 0.30 | 1 (0.91-1.098) | 1.00 | 0.856 (0.755-0.971) | **0.02** | 0.28 |
| MCPP | 1.123 (0.951-1.327) | 0.17 | 0.93 (0.814-1.062) | 0.29 | 1.004 (0.913-1.103) | 0.94 | 1.064 (0.944-1.201) | 0.31 | 0.29 |
| MEP | 1.14 (0.975-1.335) | 0.10 | 1.046 (0.913-1.198) | 0.51 | 1.035 (0.94-1.141) | 0.48 | 0.925 (0.81-1.054) | 0.24 | 0.23 |
| MEHHP | 0.889 (0.756-1.044) | 0.15 | 0.988 (0.867-1.126) | 0.86 | 1.125 (1.025-1.236) | **0.01** | 0.95 (0.835-1.081) | 0.44 | **0.04** |
| MiBP | 1.001 (0.843-1.188) | 0.99 | 0.958 (0.839-1.094) | 0.53 | 1.031 (0.938-1.132) | 0.53 | 0.912 (0.8-1.039) | 0.17 | 0.49 |
| MEOHP | 0.851 (0.723-0.999) | 0.05 | 0.898 (0.787-1.023) | 0.11 | 1.069 (0.974-1.174) | 0.16 | 0.903 (0.792-1.029) | 0.13 | **0.03** |
| MBzP | 0.932 (0.789-1.1) | 0.41 | 0.88 (0.769-1.006) | 0.06 | 1.018 (0.927-1.117) | 0.72 | 0.91 (0.802-1.031) | 0.14 | 0.28 |
| **PAH** |  |  |  |  |  |  |  |  |  |
| 1.OHN | 0.955 (0.803-1.135) | 0.60 | 1.145 (1.01-1.3) | **0.04** | 0.963 (0.888-1.044) | 0.35 | 0.971 (0.852-1.107) | 0.66 | 0.12 |
| 2.OHN | 0.97 (0.816-1.152) | 0.73 | 1.049 (0.921-1.195) | 0.47 | 0.994 (0.917-1.078) | 0.89 | 1.138 (1.003-1.292) | **0.05** | 0.30 |
| 2.OHF | 1.114 (0.926-1.343) | 0.25 | 1.108 (0.978-1.255) | 0.11 | 0.994 (0.916-1.078) | 0.89 | 1.113 (0.981-1.264) | 0.10 | 0.30 |
| 3.OHF | 1.139 (0.946-1.375) | 0.17 | 1.163 (1.029-1.316) | **0.02** | 0.991 (0.913-1.076) | 0.83 | 1.108 (0.975-1.26) | 0.12 | 0.12 |
| 1.OHPHE | 1.065 (0.907-1.252) | 0.44 | 1.034 (0.917-1.166) | 0.59 | 1.08 (0.991-1.177) | 0.08 | 1.023 (0.909-1.153) | 0.70 | 0.88 |
| 1.OHP | 1.11 (0.933-1.324) | 0.24 | 1.044 (0.933-1.168) | 0.45 | 1.047 (0.963-1.138) | 0.28 | 0.989 (0.867-1.126) | 0.86 | 0.77 |
| **VOC Metabolites** | |  |  |  |  |  |  |  |  |
| AAMA | 1.093 (0.927-1.291) | 0.29 | 1.21 (1.081-1.356) | **0.00** | 1.023 (0.942-1.112) | 0.59 | 1.126 (0.992-1.28) | 0.07 | 0.12 |
| AMCC | 1.094 (0.93-1.289) | 0.28 | 1.191 (1.061-1.339) | **0.00** | 0.968 (0.892-1.051) | 0.44 | 1.17 (1.03-1.33) | **0.02** | **0.01** |
| ATCA | 0.871 (0.755-1.004) | 0.06 | 0.871 (0.782-0.97) | **0.01** | 0.958 (0.88-1.043) | 0.33 | 0.871 (0.757-1.002) | 0.05 | 0.43 |
| BMA | 1.124 (0.961-1.316) | 0.15 | 1.112 (0.994-1.244) | 0.06 | 1.174 (1.078-1.28) | **0.00** | 1.073 (0.945-1.22) | 0.28 | 0.69 |
| NPMA | 1.089 (0.94-1.263) | 0.26 | 1.041 (0.932-1.164) | 0.47 | 1.131 (1.034-1.238) | **0.01** | 0.902 (0.796-1.021) | 0.10 | **0.03** |
| CEMA | 1.166 (0.993-1.371) | 0.06 | 1.127 (0.997-1.275) | 0.06 | 1.011 (0.933-1.096) | 0.78 | 1.167 (1.026-1.329) | **0.02** | 0.15 |
| CYMA | 1.098 (0.917-1.317) | 0.31 | 1.388 (1.238-1.557) | **0.00** | 0.941 (0.868-1.02) | 0.14 | 1.144 (0.999-1.31) | 0.05 | **0.00** |
| DHBMA | 1.046 (0.896-1.221) | 0.57 | 1.034 (0.917-1.167) | 0.59 | 1.052 (0.971-1.14) | 0.22 | 1.137 (1.001-1.292) | 0.05 | 0.71 |
| HPMA | 1.082 (0.912-1.286) | 0.37 | 1.177 (1.046-1.325) | **0.01** | 0.979 (0.907-1.058) | 0.60 | 0.933 (0.814-1.069) | 0.32 | **0.03** |
| HHPM | 1.026 (0.885-1.19) | 0.73 | 1.147 (1.013-1.299) | **0.03** | 0.974 (0.899-1.057) | 0.53 | 1.09 (0.965-1.233) | 0.17 | 0.14 |
| 2.MHA | 1.189 (1.014-1.396) | **0.03** | 1.22 (1.084-1.374) | **0.00** | 0.986 (0.909-1.069) | 0.73 | 1.066 (0.94-1.21) | 0.32 | **0.02** |
| 3.4.MHA | 1.202 (1.023-1.416) | **0.03** | 1.215 (1.078-1.372) | **0.00** | 0.992 (0.914-1.077) | 0.85 | 1.139 (1.01-1.286) | **0.03** | **0.02** |
| MHBMA3 | 0.968 (0.813-1.152) | 0.71 | 1.143 (1.014-1.29) | **0.03** | 0.911 (0.841-0.986) | **0.02** | 0.994 (0.877-1.126) | 0.92 | **0.02** |
| PGA | 0.966 (0.835-1.118) | 0.65 | 1.09 (0.967-1.232) | 0.16 | 1.019 (0.942-1.102) | 0.64 | 1.057 (0.915-1.221) | 0.45 | 0.62 |
| HPMI | 0.929 (0.78-1.105) | 0.41 | 1.131 (1.006-1.273) | **0.04** | 0.93 (0.86-1.006) | 0.07 | 0.992 (0.87-1.13) | 0.90 | **0.05** |

Odds ratios (OR) and 95% confidence intervals (CI) for associations between metabolism-disrupting agent (MDA) exposures and alcohol-related liver disease (ALD) risk, stratified by race/ethnicity. MDA concentrations were log₁₀-transformed, winsorized at the 1st and 99th percentiles, and standardized to Z-scores (mean=0, SD=1); ORs represent the change in ALD risk per 1-standard deviation increase in MDA concentration. Logistic regression models were adjusted for age, sex, body mass index (BMI), family income-to-poverty ratio (PIR), education level, smoking status, hypertension, and hyperlipidemia. All models incorporated NHANES sampling weights to account for the complex survey design. P-int represents the P-value for the multiplicative interaction term (MDA × race/ethnicity) derived from Wald tests of the interaction coefficient in logistic regression models. Urinary MDA concentrations were creatinine-corrected (ng/mg creatinine [CR]); serum PFAS concentrations are reported in ng/mL. Bold P-values indicate statistical significance (P < 0.05, two-sided).

**Table S5** BMI-Stratified Associations Between Metabolism-Disrupting Agents and Alcohol-Related Liver Disease Risk

| BMI | <25 |  | >=25 |  |  |
| --- | --- | --- | --- | --- | --- |
| Variable | OR(CI) | P value | OR(CI) | P value | P-int |
| **PFAS** |  |  |  |  |  |
| PFeDA | 1.057 (0.948-1.178) | 0.32 | 1.02 (0.946-1.099) | 0.61 | 0.59 |
| PFHxS | 1.343 (1.196-1.513) | **0.00** | 1.387 (1.285-1.499) | **0.00** | 0.66 |
| PFNA | 1.128 (1.008-1.264) | **0.04** | 1.146 (1.064-1.234) | **0.00** | 0.82 |
| **PHEN** |  |  |  |  |  |
| BP3 | 1.063 (0.955-1.184) | 0.26 | 1.056 (0.979-1.14) | 0.16 | 0.92 |
| BPA | 0.965 (0.862-1.081) | 0.54 | 0.984 (0.914-1.06) | 0.68 | 0.78 |
| MBP | 0.843 (0.756-0.941) | **0.00** | 0.803 (0.744-0.866) | **0.00** | 0.47 |
| PPB | 0.879 (0.787-0.98) | **0.02** | 0.774 (0.717-0.836) | **0.00** | 0.06 |
| 24DCP | 0.884 (0.785-0.992) | **0.04** | 0.904 (0.839-0.973) | **0.01** | 0.75 |
| 25DCP | 0.892 (0.795-1) | 0.05 | 0.899 (0.834-0.968) | **0.01** | 0.92 |
| **PHTH** |  |  |  |  |  |
| MCNP | 0.946 (0.847-1.057) | 0.33 | 1.061 (0.984-1.145) | 0.12 | 0.09 |
| MCOP | 0.975 (0.872-1.089) | 0.66 | 1.037 (0.962-1.118) | 0.34 | 0.37 |
| MECPP | 0.904 (0.808-1.011) | 0.08 | 0.985 (0.914-1.062) | 0.69 | 0.21 |
| MnBP | 0.813 (0.731-0.903) | **0.00** | 0.952 (0.881-1.027) | 0.21 | **0.02** |
| MCPP | 0.97 (0.866-1.085) | 0.60 | 0.995 (0.924-1.072) | 0.90 | 0.71 |
| MEP | 0.966 (0.863-1.08) | 0.54 | 0.946 (0.878-1.019) | 0.15 | 0.76 |
| MEHHP | 0.952 (0.852-1.063) | 0.38 | 0.977 (0.906-1.054) | 0.55 | 0.70 |
| MiBP | 0.848 (0.76-0.945) | **0.00** | 0.96 (0.89-1.035) | 0.29 | 0.07 |
| MEOHP | 0.896 (0.802-1.001) | 0.05 | 0.924 (0.856-0.996) | **0.04** | 0.66 |
| MBzP | 1.096 (0.992-1.211) | 0.07 | 0.974 (0.908-1.046) | 0.48 | 0.36 |
| **PAH** |  |  |  |  |  |
| 1.OHN | 1.096 (0.992-1.211) | 0.07 | 0.974 (0.908-1.046) | 0.48 | 0.06 |
| 2.OHN | 0.997 (0.906-1.098) | 0.96 | 0.99 (0.921-1.064) | 0.78 | 0.90 |
| 2.OHF | 1.036 (0.941-1.139) | 0.48 | 1.06 (0.986-1.14) | 0.12 | 0.70 |
| 3.OHF | 1.054 (0.958-1.159) | 0.28 | 1.096 (1.019-1.18) | **0.01** | 0.52 |
| 1.OHPHE | 1.011 (0.915-1.117) | 0.83 | 1.061 (0.988-1.139) | 0.10 | 0.44 |
| 1.OHP | 1 (0.906-1.104) | 0.99 | 1.088 (1.013-1.169) | **0.02** | 0.18 |
| **VOC Metabolites** |  |  |  |  |  |
| AAMA | 1.042 (0.949-1.144) | 0.39 | 1.14 (1.061-1.225) | **0.00** | 0.14 |
| AMCC | 1.058 (0.963-1.163) | 0.24 | 1.096 (1.02-1.177) | **0.01** | 0.56 |
| ATCA | 0.972 (0.879-1.076) | 0.59 | 0.857 (0.8-0.918) | **0.00** | **0.04** |
| BMA | 1.12 (1.017-1.235) | **0.02** | 1.068 (0.996-1.146) | 0.07 | 0.43 |
| NPMA | 1.011 (0.916-1.115) | 0.83 | 1.037 (0.968-1.112) | 0.31 | 0.67 |
| CEMA | 1.031 (0.939-1.133) | 0.52 | 1.007 (0.937-1.081) | 0.86 | 0.69 |
| CYMA | 1.091 (0.993-1.199) | 0.07 | 1.128 (1.05-1.212) | **0.00** | 0.58 |
| DHBMA | 1.027 (0.933-1.131) | 0.59 | 1.026 (0.956-1.1) | 0.48 | 0.98 |
| HPMA | 1.037 (0.942-1.141) | 0.46 | 1.002 (0.934-1.076) | 0.95 | 0.58 |
| HHPM | 1.041 (0.944-1.148) | 0.42 | 1.04 (0.97-1.115) | 0.27 | 0.99 |
| 2.MHA | 1.042 (0.945-1.149) | 0.41 | 1.142 (1.065-1.225) | **0.00** | 0.14 |
| 3.4.MHA | 1.024 (0.93-1.128) | 0.63 | 1.177 (1.097-1.264) | **0.00** | **0.02** |
| MHBMA3 | 1.022 (0.929-1.124) | 0.66 | 0.961 (0.895-1.031) | 0.27 | 0.31 |
| PGA | 0.957 (0.871-1.053) | 0.37 | 1.033 (0.963-1.108) | 0.37 | 0.21 |
| HPMI | 1.04 (0.945-1.144) | 0.43 | 0.959 (0.894-1.029) | 0.24 | 0.18 |

Odds ratios (OR) and 95% confidence intervals (CI) for associations between metabolism-disrupting agent (MDA) exposures and alcohol-related liver disease (ALD) risk, stratified by body mass index (BMI < 25 vs. ≥ 25 kg/m²). MDA concentrations were log₁₀-transformed, winsorized at the 1st and 99th percentiles, and standardized to Z-scores (mean=0, SD=1); ORs represent the change in ALD risk per 1-standard deviation increase in MDA concentration. Logistic regression models were adjusted for age, sex, race/ethnicity, family income-to-poverty ratio (PIR), education level, smoking status, hypertension, and hyperlipidemia. All models incorporated NHANES sampling weights to account for the complex survey design. P-int represents the P-value for the multiplicative interaction term (MDA × BMI) derived from Wald tests of the interaction coefficient in logistic regression models. Urinary MDA concentrations were creatinine-corrected (ng/mg creatinine [CR]); serum PFAS concentrations are reported in ng/mL. Bold P-values indicate statistical significance (P < 0.05, two-sided)

**Table S6** Hyperlipidemia-Stratified Associations Between Metabolism-Disrupting Agents and Alcohol-Related Liver Disease Risk

| Hyperlipidemia | No |  | Yes |  |  |
| --- | --- | --- | --- | --- | --- |
| Variable | OR(CI) | P value | OR(CI) | P value | P-int |
| **PFAS** |  |  |  |  |  |
| PFeDA | 0.965 (0.862-1.081) | 0.54 | 0.99 (0.922-1.063) | 0.78 | 0.71 |
| PFHxS | 1.411 (1.261-1.584) | **0.00** | 1.329 (1.233-1.435) | **0.00** | 0.39 |
| PFNA | 1.092 (0.977-1.222) | 0.12 | 1.111 (1.034-1.194) | **0.00** | 0.81 |
| **PHEN** |  |  |  |  |  |
| BP3 | 0.928 (0.828-1.04) | 0.20 | 1.102 (1.026-1.184) | **0.01** | **0.01** |
| BPA | 0.936 (0.836-1.047) | 0.25 | 1.062 (0.988-1.141) | 0.10 | 0.06 |
| MBP | 0.8 (0.717-0.892) | **0.00** | 0.828 (0.769-0.891) | **0.00** | 0.61 |
| PPB | 0.793 (0.71-0.885) | **0.00** | 0.819 (0.76-0.881) | **0.00** | 0.64 |
| 24DCP | 0.869 (0.776-0.972) | **0.02** | 0.96 (0.893-1.032) | 0.27 | 0.14 |
| 25DCP | 0.907 (0.811-1.012) | 0.08 | 0.964 (0.897-1.036) | 0.32 | 0.36 |
| **PHTH** |  |  |  |  |  |
| MCNP | 0.978 (0.878-1.088) | 0.68 | 1.138 (1.057-1.225) | **0.00** | **0.02** |
| MCOP | 1.015 (0.914-1.126) | 0.79 | 1.089 (1.011-1.174) | **0.02** | 0.28 |
| MECPP | 0.918 (0.822-1.025) | 0.13 | 1.03 (0.958-1.107) | 0.43 | 0.09 |
| MnBP | 0.872 (0.783-0.971) | **0.01** | 0.951 (0.884-1.023) | 0.18 | 0.19 |
| MCPP | 0.983 (0.883-1.094) | 0.75 | 1.045 (0.971-1.124) | 0.24 | 0.36 |
| MEP | 0.988 (0.887-1.101) | 0.83 | 0.991 (0.921-1.066) | 0.81 | 0.97 |
| MEHHP | 0.934 (0.837-1.042) | 0.22 | 1.046 (0.973-1.125) | 0.22 | 0.09 |
| MiBP | 0.843 (0.755-0.942) | **0.00** | 1.021 (0.95-1.098) | 0.57 | **0.01** |
| MEOHP | 0.884 (0.792-0.986) | **0.03** | 0.988 (0.918-1.062) | 0.74 | 0.10 |
| MBzP | 1.011 (0.911-1.121) | 0.84 | 0.97 (0.907-1.037) | 0.37 | 0.05 |
| **PAH** |  |  |  |  |  |
| 1.OHN | 1.011 (0.911-1.121) | 0.84 | 0.97 (0.907-1.037) | 0.37 | 0.51 |
| 2.OHN | 0.971 (0.875-1.077) | 0.58 | 1.048 (0.98-1.12) | 0.17 | 0.23 |
| 2.OHF | 0.979 (0.882-1.086) | 0.69 | 1.068 (0.998-1.142) | 0.06 | 0.17 |
| 3.OHF | 0.995 (0.897-1.103) | 0.93 | 1.077 (1.007-1.153) | **0.03** | 0.21 |
| 1.OHPHE | 0.991 (0.895-1.097) | 0.86 | 1.085 (1.014-1.162) | **0.02** | 0.14 |
| 1.OHP | 0.954 (0.862-1.056) | 0.36 | 1.101 (1.029-1.179) | **0.01** | **0.02** |
| **VOC Metabolites** |  |  |  |  |  |
| AAMA | 0.976 (0.884-1.076) | 0.62 | 1.174 (1.097-1.256) | **0.00** | **0.00** |
| AMCC | 0.988 (0.893-1.094) | 0.82 | 1.092 (1.023-1.167) | **0.01** | 0.10 |
| ATCA | 0.851 (0.77-0.939) | **0.00** | 0.928 (0.868-0.992) | **0.03** | 0.15 |
| BMA | 0.988 (0.895-1.091) | 0.81 | 1.179 (1.103-1.261) | **0.00** | **0.00** |
| NPMA | 0.984 (0.891-1.087) | 0.75 | 1.08 (1.011-1.155) | **0.02** | 0.13 |
| CEMA | 1.021 (0.923-1.13) | 0.68 | 1.054 (0.987-1.126) | 0.12 | 0.61 |
| CYMA | 1.031 (0.934-1.139) | 0.54 | 1.087 (1.017-1.162) | **0.01** | 0.39 |
| DHBMA | 0.987 (0.894-1.09) | 0.79 | 1.089 (1.019-1.164) | **0.01** | 0.11 |
| HPMA | 0.974 (0.879-1.079) | 0.61 | 1.019 (0.954-1.088) | 0.57 | 0.47 |
| HHPM | 0.943 (0.85-1.045) | 0.26 | 1.075 (1.007-1.147) | **0.03** | **0.04** |
| 2.MHA | 1.005 (0.908-1.113) | 0.93 | 1.105 (1.035-1.18) | **0.00** | 0.12 |
| 3.4.MHA | 1.007 (0.912-1.113) | 0.89 | 1.127 (1.055-1.204) | **0.00** | 0.07 |
| MHBMA3 | 0.969 (0.876-1.073) | 0.55 | 0.965 (0.904-1.031) | 0.29 | 0.94 |
| PGA | 0.942 (0.854-1.038) | 0.23 | 1.05 (0.982-1.122) | 0.15 | 0.07 |
| HPMI | 0.968 (0.874-1.072) | 0.54 | 0.968 (0.907-1.034) | 0.34 | 1.00 |

Odds ratios (OR) and 95% confidence intervals (CI) for associations between metabolism-disrupting agent (MDA) exposures and alcohol-related liver disease (ALD) risk, stratified by hyperlipidemia status. MDA concentrations were log₁₀-transformed, winsorized at the 1st and 99th percentiles, and standardized to Z-scores (mean=0, SD=1); ORs represent the change in ALD risk per 1-standard deviation increase in MDA concentration. Logistic regression models were adjusted for age, sex, race/ethnicity, body mass index (BMI), family income-to-poverty ratio (PIR), education level, smoking status, and hypertension. All models incorporated NHANES sampling weights to account for the complex survey design. P-int represents the P-value for the multiplicative interaction term (MDA × hyperlipidemia) derived from Wald tests of the interaction coefficient in logistic regression models. Urinary MDA concentrations were creatinine-corrected (ng/mg creatinine [CR]); serum PFAS concentrations are reported in ng/mL. Bold P-values indicate statistical significance (P < 0.05, two-sided).

**Table S7** Cross-Validation Performance Comparison of Machine Learning Algorithms for Alcohol-Related Liver Disease Risk Prediction

| **Model** | **Fold** | **AUC** | **Mean** | **SD** |
| --- | --- | --- | --- | --- |
| Logistic | Fold1 | 0.750 | 0.748 | 0.013 |
|  | Fold2 | 0.706 |  |  |
|  | Fold3 | 0.786 |  |  |
|  | Fold4 | 0.738 |  |  |
|  | Fold5 | 0.758 |  |  |
| ENET | Fold1 | 0.721 | 0.738 | 0.017 |
|  | Fold2 | 0.700 |  |  |
|  | Fold3 | 0.787 |  |  |
|  | Fold4 | 0.714 |  |  |
|  | Fold5 | 0.767 |  |  |
| DT | Fold1 | 0.616 | 0.676 | 0.026 |
|  | Fold2 | 0.615 |  |  |
|  | Fold3 | 0.739 |  |  |
|  | Fold4 | 0.685 |  |  |
|  | Fold5 | 0.722 |  |  |
| RF | Fold1 | 0.749 | 0.756 | 0.018 |
|  | Fold2 | 0.693 |  |  |
|  | Fold3 | 0.793 |  |  |
|  | Fold4 | 0.755 |  |  |
|  | Fold5 | 0.788 |  |  |
| XGBoost | Fold1 | 0.751 | 0.750 | 0.019 |
|  | Fold2 | 0.681 |  |  |
|  | Fold3 | 0.770 |  |  |
|  | Fold4 | 0.756 |  |  |
|  | Fold5 | 0.792 |  |  |
| SVM | Fold1 | 0.742 | 0.751 | 0.010 |
|  | Fold2 | 0.732 |  |  |
|  | Fold3 | 0.786 |  |  |
|  | Fold4 | 0.738 |  |  |
|  | Fold5 | 0.758 |  |  |
| MLP | Fold1 | 0.702 | 0.738 | 0.014 |
|  | Fold2 | 0.729 |  |  |
|  | Fold3 | 0.772 |  |  |
|  | Fold4 | 0.719 |  |  |
|  | Fold5 | 0.768 |  |  |
| LightGBM | Fold1 | 0.762 | 0.760 | 0.020 |
|  | Fold2 | 0.685 |  |  |
|  | Fold3 | 0.786 |  |  |
|  | Fold4 | 0.766 |  |  |
|  | Fold5 | 0.801 |  |  |
| KNN | Fold1 | 0.720 | 0.715 | 0.007 |
|  | Fold2 | 0.716 |  |  |
|  | Fold3 | 0.739 |  |  |
|  | Fold4 | 0.697 |  |  |
|  | Fold5 | 0.702 |  |  |

Area under the receiver operating characteristic curve (AUC) values for nine machine learning algorithms evaluated via 5-fold cross-validation on the training set. The dataset was randomly split 7:3 into training and test sets with stratified sampling to maintain the proportion of ALD cases. Feature selection was performed independently within each fold using the intersection of features identified by both LASSO regression and Random Forest, yielding 14 consensus features: NPMA, BMA, AMCC, MCOP, HHPM, ATCA, 1-OHPhe, MEOHP, HPMI, 2-OHN, age, BMI, sex, and hyperlipidemia. Each algorithm was trained and evaluated on the five folds using grid-search hyperparameter optimization. AUC represents the discrimination performance for each individual fold; Mean represents the average AUC across the five folds; SD (standard deviation) quantifies the variability in performance across folds. LightGBM achieved the highest mean cross-validation AUC (0.760, SD=0.020) and was selected as the final model for subsequent evaluation on the held-out test set (AUC=0.706, 95% CI: 0.690-0.723).

**Table S8** Performance Metrics of Machine Learning Algorithms on the Held-Out Test Set

| **Model** | **Logistic** | **ENET** | **DT** | **RF** | **XGBoost** | **SVM** | **MLP** | **LightGBM** | **KNN** |
| --- | --- | --- | --- | --- | --- | --- | --- | --- | --- |
| accuracy | 0.634 | 0.624 | 0.679 | 0.638 | 0.620 | 0.655 | 0.620 | 0.659 | 0.568 |
| kap | 0.264 | 0.262 | 0.339 | 0.288 | 0.244 | 0.307 | 0.242 | 0.312 | 0.159 |
| sens | 0.648 | 0.528 | 0.786 | 0.547 | 0.656 | 0.660 | 0.597 | 0.673 | 0.447 |
| spec | 0.617 | 0.742 | 0.547 | 0.750 | 0.591 | 0.648 | 0.648 | 0.641 | 0.719 |
| ppv | 0.678 | 0.718 | 0.683 | 0.731 | 0.564 | 0.700 | 0.679 | 0.699 | 0.664 |
| npv | 0.585 | 0.559 | 0.673 | 0.571 | 0.681 | 0.606 | 0.565 | 0.612 | 0.511 |
| mcc | 0.264 | 0.274 | 0.344 | 0.300 | 0.246 | 0.307 | 0.245 | 0.312 | 0.170 |
| j_index | 0.265 | 0.270 | 0.333 | 0.297 | 0.247 | 0.309 | 0.246 | 0.314 | 0.165 |
| bal_accuracy | 0.632 | 0.635 | 0.667 | 0.649 | 0.624 | 0.654 | 0.623 | 0.657 | 0.583 |
| detection_prevalence | 0.530 | 0.408 | 0.638 | 0.415 | 0.519 | 0.523 | 0.488 | 0.533 | 0.373 |
| precision | 0.678 | 0.718 | 0.683 | 0.731 | 0.564 | 0.700 | 0.679 | 0.699 | 0.664 |
| recall | 0.648 | 0.528 | 0.786 | 0.547 | 0.656 | 0.660 | 0.597 | 0.673 | 0.447 |
| f_meas | 0.662 | 0.609 | 0.731 | 0.626 | 0.606 | 0.680 | 0.635 | 0.686 | 0.534 |
| roc_auc | 0.670 | 0.676 | 0.684 | 0.712 | 0.696 | 0.692 | 0.664 | 0.706 | 0.635 |

Comprehensive performance evaluation of nine machine learning algorithms on the independent test set (n=4,042, 30% of total sample). All models were trained on the training set (n=9,430) using 14 consensus features identified through nested cross-validation (NPMA, BMA, AMCC, MCOP, HHPM, ATCA, 1-OHPhe, MEOHP, HPMI, 2-OHN, age, BMI, sex, hyperlipidemia). The test set was completely isolated from feature selection and model training procedures to provide unbiased performance estimates. Performance metrics include: accuracy (overall classification accuracy); kap (Cohen's kappa coefficient, chance-corrected agreement); sens (sensitivity/recall, true positive rate); spec (specificity, true negative rate); ppv (positive predictive value/precision); npv (negative predictive value); mcc (Matthews correlation coefficient, balanced measure accounting for class imbalance); j_index (Youden's index, sens + spec - 1); bal_accuracy (balanced accuracy, average of sensitivity and specificity); detection_prevalence (proportion of positive predictions); precision (identical to ppv); recall (identical to sensitivity); f_meas (F1-score, harmonic mean of precision and recall); roc_auc (area under the receiver operating characteristic curve). LightGBM achieved the highest AUC (0.706, 95% CI: 0.690-0.723) and balanced accuracy (0.657), demonstrating optimal discrimination performance and was selected as the final model.

**Figure S1** Flowchart of Participant Selection from NHANES 2005-2006, 2011-2012, 2013-2014, and 2015-2016

Participant selection process for the study examining associations between metabolism-disrupting agents (MDAs) and alcohol-related liver disease (ALD) risk. From an initial pool of 39,156 participants across four NHANES cycles (2005-2006, 2011-2012, 2013-2014, 2015-2016), participants were sequentially excluded based on the following criteria: (1) age <20 or >85 years, pregnancy, or lactation; (2) missing essential data on anthropometrics (height, weight, waist circumference), alcohol consumption, blood pressure, or laboratory tests (ALT, AST, HDL, glucose, triglycerides); (3) presence of other liver diseases including metabolic dysfunction-associated steatotic liver disease (MASLD), viral hepatitis B or C, or other unspecified liver conditions. The final analytical sample comprised 13,472 participants (6,164 with ALD and 7,308 without ALD) who had complete data on all study variables and MDA biomarker measurements.

Abbreviations: ALT, alanine aminotransferase; AST, aspartate aminotransferase; HDL, high-density lipoprotein cholesterol.

**Figure S2** Feature Selection Process Using LASSO Regression and Random Forest for Alcohol-Related Liver Disease Risk Prediction

Identification of consensus features for machine learning model development through dual feature selection methods. LASSO (Least Absolute Shrinkage and Selection Operator) regression with L1 regularization for variable selection. The left panel shows the cross-validation curve for tuning parameter (λ) selection, with binomial deviance plotted against log(λ). The optimal λ value (indicated by the vertical dashed line) was selected based on the minimum cross-validation error plus one standard error (1-SE rule) to balance model complexity and predictive performance. The right panel displays the coefficient paths for all 40 MDAs and covariates as a function of log(λ), with each colored line representing one variable. As λ increases, coefficients shrink toward zero, with less important features being eliminated first. Random Forest feature importance ranking based on mean decrease in Gini impurity. Variables are ranked by their importance scores, with higher values indicating greater contribution to classification accuracy. The model was trained with 1000 decision trees. Features selected by both LASSO (non-zero coefficients at optimal λ) and Random Forest (above empirical importance threshold) were retained as consensus features for subsequent machine learning model development. This intersection approach yielded 14 consensus features: NPMA, BMA, AMCC, MCOP, HHPM, ATCA, 1-OHPhe, MEOHP, HPMI, 2-OHN, age, BMI, sex, and hyperlipidemia. Feature selection was performed independently within each fold of 5-fold cross-validation to prevent data leakage and ensure unbiased model evaluation.

**Figure S3 Dose-Response Relationships Between Additional Metabolism-Disrupting Agents and Alcohol-Related Liver Disease Risk**

Restricted cubic spline (RCS) curves illustrating dose-response associations between 24 additional metabolism-disrupting agent (MDA) exposures and alcohol-related liver disease (ALD) risk in 13,472 NHANES participants (2005-2006, 2011-2012, 2013-2014, 2015-2016). This figure complements Figure 1 by presenting RCS analyses for MDAs not featured in the main text. MDA concentrations were log₁₀-transformed, winsorized at the 1st and 99th percentiles, standardized to Z-scores (mean=0, SD=1), and modeled continuously using RCS with four knots positioned at the 5th, 35th, 65th, and 95th percentiles of the standardized exposure distribution. The reference level (OR=1.0) was set at the 10th percentile of each MDA's distribution. Logistic regression models were adjusted for age, sex, race/ethnicity, body mass index (BMI), family income-to-poverty ratio (PIR), education level, smoking status, hypertension, and hyperlipidemia, incorporating NHANES sampling weights to generate nationally representative estimates. The x-axis represents standardized MDA concentrations (Z-scores); the y-axis represents odds ratios (OR) for ALD risk. Solid lines represent point estimates of ORs, and shaded areas indicate 95% confidence intervals. Histograms at the bottom of each panel display the distribution of standardized MDA concentrations across the study population. P-overall indicates statistical significance of the overall association; P-nonlinear tests for departure from linearity derived from Wald tests of the cubic spline terms. Original urinary MDA concentrations were creatinine-corrected (ng/mg creatinine); serum PFAS concentrations were in ng/mL prior to log-transformation and standardization.

**Figure S4** Identification of 192 Shared Genes Between Metabolism-Disrupting Agent Targets and Alcohol-Related Liver Disease-Related Genes

Venn diagram illustrating the overlap between computationally predicted metabolism-disrupting agent (MDA) protein targets and alcohol-related liver disease (ALD)-related genes identified through transcriptomic analysis. MDA protein targets were predicted using Swiss Target Prediction (STP, [http://swisstargetprediction.ch/](http://swisstargetprediction.ch/) ) and Similarity Ensemble Approach (SEA, [https://sea.bkslab.org/](https://sea.bkslab.org/) ) databases, with the union of targets from both databases constituting the MDA target set. ALD-related genes were identified from Gene Expression Omnibus (GEO) dataset GSE28619 (liver tissue samples from 7 healthy controls and 15 ALD patients) through two complementary approaches: (1) differential expression analysis using the limma package identified differentially expressed genes (DEGs) at thresholds of P < 0.05 and |log₂ fold-change| > 1.2; (2) weighted gene co-expression network analysis (WGCNA) identified gene modules significantly correlated with ALD phenotype (soft-thresholding power β selected to achieve scale-free topology R² > 0.85). The union of DEGs and WGCNA module genes formed the ALD-related gene set. The intersection yielded **192 shared genes** representing candidate mechanistic mediators linking MDA exposures to ALD pathogenesis. Gene symbols are displayed in the overlapping region of the diagram. These 192 genes were subjected to Gene Ontology (GO) and KEGG pathway enrichment analyses (Figure 3B-C) to elucidate potential biological mechanisms underlying MDA-ALD associations.
